# A randomized trial of adapted versus standard versions of the Transdiagnostic Intervention for Sleep and Circadian Dysfunction implemented via facilitation and delivered by community mental health providers: improving the “fit” of psychological treatments by adapting to context

**DOI:** 10.1186/s13012-025-01440-9

**Published:** 2025-07-09

**Authors:** Allison G. Harvey, Emma R. Agnew, Rafael Esteva Hache, Julia M. Spencer, Marlen Diaz, Estephania Ovalle Patino, Anne Milner, Lu Dong, Amy M. Kilbourne, Daniel J. Buysse, Catherine A. Callaway, Laurel D. Sarfan

**Affiliations:** 1 Department of Psychology, University of California, 2121 Berkeley Way, Berkeley, CA 94720 US; 2 Department of Learning Health Sciences, University of Michigan Medical School, Ann Arbor, MI USA; 3 RAND Corporation, Santa Monica, CA US; 4 Office of Research and Development, Veterans Health Administration, U.S. Department of Veterans Affairs, DC, MI US; 5 University of Pittsburgh, Pittsburgh, PA US; 6 Department of Research and Evaluation, Kaiser Permanente Southern California, Pasadena, CA US

**Keywords:** Adaptation, Facilitation, Integrated Promoting Action on Research Implementation in Health Services framework (i-PARIHS), Replicating Effective Programs framework (REP), Community mental health, Mental illness, Sleep, Circadian, Insomnia, Transdiagnostic, Psychosis, Depression, Anxiety disorder, Bipolar disorder

## Abstract

**Background:**

Grounded in the Integrated Promoting Action on Research Implementation in Health Services framework (i-PARIHS) and the Replicating Effective Programs framework (REP), the goal is to determine if the use of theory, data and end-user perspectives to guide an adaptation of the Transdiagnostic Intervention for Sleep and Circadian Dysfunction (TSC) yields better outcomes and improves the “fit” of TSC to community mental health centers (CMHCs), relative to the standard version.

**Methods:**

Ten counties in California were cluster-randomized by county to Adapted or Standard TSC. Within each county, adults who exhibited sleep and circadian dysfunction and serious mental illness (SMI) were randomized to immediate TSC or Usual Care followed by Delayed Treatment with TSC (UC-DT). Facilitation was the implementation strategy. The participants were 93 CMHC providers who delivered TSC (Standard = 30; Adapted = 63) and 396 CMHC patients (Standard = 74; Adapted = 124; UC-DT = 198). Patient assessments were completed at pre-treatment, post-treatment, and six months after treatment (6FU). Provider assessments were completed at post-training, mid-treatment, and post-treatment.

**Results:**

TSC (combining Adapted and Standard), relative to UC-DT before delayed treatment with TSC, was associated with improvement from pre- to post-treatment in sleep disturbance (*b* = -10.91, *p* < 0.001, *d* = -1.52), sleep-related impairment (*b* = -9.52, *p* < 0.001, *d* = -1.06), sleep health composite (*b* = 1.63, *p* < 0.001, *d* = 0.95), psychiatric symptoms (*b* = -6.72, *p* < 0.001, *d* = -0.52), and overall functional impairment (*b* = -5.12, *p* < 0.001, *d* = -0.71). TSC’s benefits for functional impairment and psychiatric symptoms were mediated by improvements in sleep and circadian problems. Adapted versus Standard TSC did not differ on provider ratings of fit and better fit did not mediate the relation between TSC condition and patient outcome.

**Conclusions:**

TSC can be delivered by CMHC providers. Adapted and Standard TSC both fit the CMHC context. These findings are interpreted through the lens of the four core constructs of the i-PARIHS framework.

**Trial registration:**

Clinicaltrials.gov identifier: NCT04154631. Registered on November 6, 2019. https://clinicaltrials.gov/ct2/show/NCT04154631

**Supplementary Information:**

The online version contains supplementary material available at 10.1186/s13012-025-01440-9.

Contributions to the literature

- Research on whether new treatments fit diverse practice settings is crucial, as poor fit may prevent sustained use.
- Following the REP framework, theory, data, and perspectives from end-users informed the adaptation of a transdiagnostic sleep treatment to improve its fit to community mental health centers (CMHCs).
- Using external facilitation as the implementation strategy, within the i-PARIHS framework, CMHC providers were trained to deliver the Adapted or Standard version of the sleep treatment to CMHC patients with sleep problems and serious mental illness.
- Both treatments improved sleep, mental health and functioning and were rated by providers as fitting with the CMHC context.

## Background

A critical barrier to expanding access to evidence-based psychological treatments (EBPTs) is the potential mismatch between research settings, in which EBPTs are typically developed, and routine practice settings, in which EBPTs are ultimately meant to be delivered. This can result in a poor “fit” between the EBPT and the routine practice setting. Strong fit is defined as the “match between the strategies, procedures, or elements of an intervention and the values, needs, skills, and resources available in a setting” (p. 1) [1]. Fit is crucial because there is evidence that many routine practice settings that initially adopted EBPTs cannot sustain them, in part due to poor fit (e.g., [2, 3]). Indeed, EBPT fit directly predicts a range of implementation [4] and sustainment outcomes (e.g., [5, 6]) and is often included in implementation science frameworks (e.g., [7]). Along the path to expand access to EBPTs, one goal of the present study was to test a replicable and generalizable approach to maximize fit to routine practice settings.

This study was grounded in the Integrated Promoting Action on Research Implementation in Health Services (i-PARIHS) framework [8]. i-PARIHS was selected because of its “overarching view of implementation as iterative, negotiated and relational” (p. 9) [8]. These features were highly advantageous given the recipients and context for the present study. According to i-PARIHS, the successful implementation of an EBPT into practice is a function of the quality of evidence for the innovation, the recipients of the innovation, the characteristics of the context into which the innovation will be implemented, and the approach by which the innovation is integrated or facilitated into the context. As outlined by the i-PARIHS framework, Table 1 presents a summary of the innovation components, recipients, contextual factors, and the approach to facilitation that comprised the current study and that will now be explained.

**Table 1:** Overview of i-PARIHS Core Constructs, Background Considerations, and Key Findings

i-PARIHS core constructs	Background Considerations	Key Findings from the Present Study
Innovation		
Efficacy data for TSC	The standard version of TSC had been associated with improvements in outcomes, when delivered by providers employed in an academic setting	TSC is superior to usual care. Also, the results extend knowledge in two ways: the providers of TSC were employed in CMHC contexts and an Adapted and Standard version of TSC yielded positive outcomes
Consideration of the characteristics of TSC that impact uptake	While engaging with community partners, there was a clear need and preference for treatments with improved feasibility	The length and complexity of Standard TSC may have contributed to the lower recruitment rates and higher drop-out, compared to Adapted TSC
Aligning evidence with local priorities and practice	Adapted TSC was designed to fit with local needs, including fewer and shorter sessions and trainings	Provider ratings of the fit and credibility of Adapted TSC did not differ from Standard TSC
Recipient		
Recipients of the innovation: People diagnosed with SMI	In a prior qualitative study, concerns were raised about potential cognitive overload experienced by patients who received the standard version of TSC	Patient improvements were observed in sleep, psychiatric symptoms and functional impairment at the post-treatment assessment. Improvements in psychiatric symptoms and functional impairment were mediated through the proposed mechanism of change – namely, sleep and circadian functioning
Recipients involved in implementation: Providers who deliver TSC	In a prior qualitative study, concerns were raised about the fit between the standard version of TSC and the high workload of providers	Providers in both conditions rated TSC as acceptable, appropriate and feasible
Context		
Local level (micro)	The micro level was the main focus of facilitation. The type and intensity of facilitation varied across providers and sites. Example activity: Establishing CE credits for participating in training and to help providers meet license requirements	Findings for the present study focused on the innovation and recipient levels
Organizational level (meso)	Example activities: organization-wide trainings; establishing relationships with leadership; email listserve	As above
Outer context/Wider health system (macro)	Example activity: efforts to promote sleep health as essential for mental health	As above
Facilitation		
External facilitation, supported by project leadership	Facilitation is the flexible and responsive use of an integrated set of evidence-based implementation strategies and tailoring these strategies to meet the specific needs of the recipients and the context	Facilitation was effective in supporting providers to deliver TSC. Facilitation was well suited to the variety of unique challenges and obstacles faced by each provider and at each site

i-PARIHS core constructs are derived from Harvey & Kitson’s theoretical papers [8, 26]

### Innovation

The EBPT for this project was the Transdiagnostic Intervention for Sleep and Circadian Dysfunction (TSC)^1^ [9]. TSC is grounded in, and aims to improve, each dimension of the Sleep Health Framework [10]. TSC was designed to address the sleep and circadian dysfunction most frequently experienced by people diagnosed with serious mental illness (SMI). Treating sleep and circadian dysfunction is an important domain when caring for people diagnosed with SMI as poor sleep and circadian dysregulation predicts and predates the onset and worsening of SMI symptoms, as well as poorer mental (e.g., [11, 12]) and physical health (e.g., [13]). Furthermore, sleep and circadian problems are modifiable (e.g., [14–16]). In a prior trial that included 121 SMI patients, the standard version of TSC was associated with sustained improvements in sleep and circadian problems, functional impairment, and psychiatric symptoms, relative to usual care [15]. In selecting TSC for the current study, we considered the characteristics of the TSC that are implementable, which is foundational to i-PARIHS. Here are three examples. First, an important feature of the sample included in the prior trial of TSC included that fewer than 10% of the participants met criteria for *only one* full sleep or circadian diagnosis. Also, more than 80% of people met criteria for *at least one* subdiagnostic sleep or circadian problem. Furthermore, meeting criteria for more full and/*or* subdiagnostic sleep and circadian comorbidities was associated with worse overall impairment [17]. Nonetheless, people with these complex sleep and circadian problems responded well to TSC [15], perhaps because TSC offers one treatment that is specifically designed to be useful across various sleep and circadian problems and across various mental illness diagnoses, as opposed to offering many single disorder treatments for each specific problem and diagnosis [9]. Second, a moderator of the results in the prior trial indicated that participants who were African American or Black were particularly responsive to TSC [18], perhaps because the individualized case formulation driven approach increased engagement with and the relevance of the intervention. Third, we collected qualitative data from providers [19] and the SMI patients [20] to learn about the fit of TSC to the routine practice setting. The findings highlighted the high workload of providers and the cognitive overload experienced by patients who received the standard version of TSC. Together, these three sets of findings bode well for TSC as an innovation that is suitable for implementation. However, we hypothesize that the standard version of TSC, which is delivered in eight 50-min sessions, might “affect its migration and uptake in different settings” (p. 4) [8] and that there may be a need to align the evidence with local priorities and practice (per i-PARIHS), a point to which we will return below.

### Recipients

The study described herein includes two groups of recipients of the innovation (TSC), another key domain of i-PARIHS. The first group of recipients of the innovation are SMI patients who are typically experiencing comorbid sleep and circadian problems and comorbid mental health challenges. Aim 1 of this report will focus on the outcomes for these recipients of TSC. The second group of recipients for this study were the routine practice providers who delivered TSC to the SMI patients. We selected this group following the National Institute of Health's Stage Model [21]. Specifically, while in the prior trial the providers of TSC were employed, trained, and supervised within an academic setting, for the present study we took the critical next step; namely, we tested TSC when it was delivered by providers who were employed, trained and supervised within the routine practice setting. Aim 2 of this report will focus on provider perspectives on key aspects of their experience of delivering TSC.

### Context

i-PARIHS encourages recognition of various levels of context (see Table 1). The context for the present study was a network of community mental health centers (CMHCs). In the United States, CMHCs are large providers of affordable mental health services for people who are low-income and diverse with respect to demographic and clinical presentations. At the local (micro) level, the CMHC providers have insufficient time and resources, carry a heavy caseload, and the patients served experience high rates of comorbidity and complexity [22–24]. Also, it can be difficult for CMHC providers to receive adequate training and supervision in EBPTs [25]. However, the wellness and recovery philosophy of CMHCs—along with a strong emphasis on skill-building, education, self-management, and promoting recovery—align closely with TSC. The CMHC providers who delivered TSC work within clinics under the governance of a leadership team (meso-level contextual factors) which are embedded within the administrative structures that fund the employees and services, pay for services delivered and that maintain the physical sites (macro-level contextual factors).

### Facilitation

Moving onto the final essential element within i-PARIHS, facilitation “that implementation through assessing and responding to characteristics of the innovation and the recipients” (p. 6) [8]. Facilitation encompasses both the individuals who facilitate and the methods they employ to promote the adoption and long-term sustainability of innovations. More specifically, facilitation involves the flexible and responsive use of an integrated set of implementation strategies and tailoring these strategies to meet the specific needs of the recipients and the context. The use of adaptive implementation strategies reflects that a one-size-fits-all approach may not be effective in complex settings like CMHCs [26]. In one systematic review of implementation in primary care, new innovations implemented with facilitation were 2.76 times more likely to be adopted [27]. The present study used external facilitation to support the implementation of TSC into CMHCs. The external facilitators, employed within the university setting, played a crucial role in guiding the complex change processes and challenges, ensuring the implementation processes align with the policies and values of the CMHCs and engaging in the problem solving needed to implement TSC into existing workflows, address recipients'motivations, and adapt implementation to the micro, meso and macro contexts of the CMHCs [28, 29].

### Fitting TSC to the recipients and the context

Respecting the “relational dynamism” among the core elements of i-PARIHS (p. 23) [30] as well as the feedback from SMI patients [20] and CMHC providers [19], we undertook a process to improve the fit of TSC to the recipients and the context for this implementation effort. Fit is particularly important for resource-constrained practice settings, such as CMHCs. Further, according to i-PARIHS, “people rarely take the original form … and apply it within an implementation project but rather they incorporate evidence in a number of different ways, which typically involves adapting the original evidence” (p. 4) [8]. Taken together, the 8 × 50 min standard alone treatment sessions that comprise the standard version of TSC may not be a fit for the existing practice and values of the CMHC context. Thus, in this study, an Adapted version of TSC was developed and compared to Standard TSC. Adapted TSC is hypothesized to increase the compatibility of TSC with the recipients and the context due to its concise, structured format tailored for time-constrained providers and its focus on delivering the fundamental principles of sleep health to all recipients.

Indeed, Chambers et al. [31] has cautioned against “creating and freezing an intervention” as this reduces the customization and optimization that are the basis of effective sustainment (p. 2). How might an EBPT be customized and optimized to fit a specific context? The answer most certainly involves theory, data, and end-user input [15, 32, 33]. The development and testing of Adapted TSC in this study created an opportunity to empirically test the implementation strategy of “promoting adaptability” [34]. In other words, does this strategy of promoting adaptability—defined as “identify[ing] the ways a clinical innovation can be tailored to meet local needs and clarify[ing] which elements of the innovation must be maintained to preserve fidelity”—enhance implementation and clinical outcomes? As described in the protocol paper [35], the Replicating Effective Programs (REP) framework [36] was selected as the theoretical basis for the adaptation process that resulted in Adapted TSC. Phase 1 of REP (Pre-Condition) was completed prior to the present study. This involved several elements. First, we established that there is a need for an effective, feasible EBPT for SMI in CMHCs and that sleep and circadian functioning was a target that could help address this need [15]. Second, we established that TSC in CMHCs has empirical support [15]. Third, we used the implementation strategy of identifying barriers and facilitators to gather perspectives from CMHC staff and SMI patients on the fit and packaging of TSC [19, 20]. In this process, both the dose and complexity of Standard TSC were considered to be barriers to the implementation of TSC [19]. Fourth, a prior study identified the TSC treatment skills that were most utilized by patients. [37] The associated treatment elements were retained as fixed components of Adapted TSC. Fifth, we considered TSC’s theoretical underpinnings and mechanisms of action. [9, 10] Treatment strategies that addressed the key mechanisms were also retained as fixed components [33, 38, 39]. Sixth, we piloted Adapted TSC with 21 adults through the PI’s UC Berkeley research clinic (unpublished data). Feedback from the providers and the patients were used to further refine Adapted TSC. In Phase 2 of REP (Pre-Implementation), based on feedback from CMHC leadership, staff, and patients, we tailored the delivery of TSC training and therapy materials to the CMHC setting [19, 20]. Throughout REP Phases 1 and 2, following leading adaptation frameworks, we sought to ensure that Adapted TSC would be relevant to the broadest range of patients and to account for factors that impact implementation (e.g., resources required) [33, 40, 41]. Collectively, these efforts addressed Phases 1 and 2 of the REP framework. The current study aims to address Phase 3 (Implementation) of REP. Following prior research in CMHCs [42], Adapted TSC involved a modular design, with 5 fixed core modules that address the key mechanisms of change. There is also one optional module that is delivered only when a patient is experiencing sleep-related worry. Given the CMHC context, the general strategy was to remove all excess treatment elements to provide the most simple and efficient version of TSC. Beyond these fixed components, providers in both treatment arms were told that they could make fidelity-consistent ‘soft’ adaptations in how they delivered the fixed intervention components in ways that felt relevant and accessible for patients [43]. For instance, instead of using examples offered in the treatment manual, providers were encouraged to use examples that were relevant to their specific client. As another example, if the language in the treatment manual seemed too complex for a given client, providers were encouraged to simplify using their own wording.

### The present study

The overarching goal of this study was to evaluate whether manipulating the fit of TSC to the CMHC context (Adapted TSC) predicts better implementation outcomes relative to an original version of TSC (Standard TSC). This study is a hybrid type 2 effectiveness-implementation study [44] conducted in the CMHCs of counties across California in the United States. This report is focused on Parts 1 of three parts. Part 1 is the Implementation Phase, during which TSC was implemented in CMHCs via facilitation [35]. Parts 2 and 3 will be reported in subsequent papers. Part 2, is the Train-the-Trainer Phase, during which CMHC providers learn to train and supervise their peers in the delivery of TSC [45]. Part 3 is the Sustainment Phase, during which we will assess the extent to which TSC activities are sustained after facilitation has ceased [46].

As described elsewhere [35], all CMHC sites were cluster-randomized by county to Adapted TSC or Standard TSC with 1:1 allocation. Then, within each county, patients were randomized to immediate TSC or UC-DT. TSC was delivered by CMHC providers.

For the present study, the first aim was to assess the effectiveness of TSC, compared to UC-DT. Note that the assessment points for the latter were completed before and after usual care and before delayed treatment with TSC. We hypothesized that compared to UC-DT, TSC (combined Adapted and Standard) would be associated with larger reductions in the primary patient outcome of sleep disturbance and the secondary patient outcomes of sleep-related impairment, overall sleep health, functional impairment, and psychiatric symptoms. We also hypothesized that TSC’s benefits for functional impairment and psychiatric symptoms would be mediated by improvements in sleep and circadian problems. This aim is important because the prior evidence for Standard TSC comes from a study in which university-based providers administered the treatment [15]. In contrast, CMHC providers delivered TSC in the present study. Therefore, this aim addresses the fundamental question of how outcomes from TSC (both Adapted and Standard) performed when implemented by community providers. The second aim was to evaluate whether TSC treatment condition (Adapted versus Standard TSC) is associated with fit to the CMHC context, operationalized as provider ratings of acceptability, feasibility, and appropriateness. We hypothesized that Adapted TSC would be superior to Standard TSC with respect to the primary provider outcome of acceptability and the secondary provider outcomes of feasibility and appropriateness. The third aim was to evaluate whether better fit mediates the relation between TSC treatment condition and patient outcome. We hypothesized that relative to Standard TSC, Adapted TSC would be associated with greater reductions in the primary and secondary patient outcomes indirectly through higher provider ratings of acceptability, feasibility, and appropriateness. Exploratory analyses sought to: (1) compare Adapted and Standard TSC on patient perceptions of credibility/improvement, and select PhenX Toolkit outcomes; and (2) determine whether treatment effects are moderated by risk factors including age, sex, sleep symptoms, impairment and psychiatric symptoms at baseline (e.g., [18]).

## Method

### Setting and participants

Community health center sites in the following ten counties in California, USA participated: Alameda, Contra Costa, Kings, Monterey, Placer, Santa Cruz, Solano, Santa Clara, Santa Barbara, and Lake. The participants were 93 CMHC providers (Standard TSC = 30; Adapted TSC = 63) and 396 CMHC patients.^2^ Of the patients, 198 were randomized to receive TSC immediately (Standard TSC = 74; Adapted TSC = 124) and 198 were randomized to UC-DT. The larger number of participants in Adapted TSC resulted from stronger recruitment in the counties cluster randomized to this condition, as compared to the Standard TSC condition. The inclusion criteria for selecting the CMHC sites within counties from which to recruit providers and patients were: (1) provision of publicly funded adult mental health outpatient services and (2) support from CMHC leadership.

CMHCs preferred to determine which providers were eligible to receive TSC training at each site (e.g., case managers, nurses, psychiatrists), because this aligns with their real-world practice. The other inclusion criteria for providers were: (1) employed or able to deliver client-facing services to patients within the CMHC; (2) interested in learning and delivering TSC; and (3) volunteered to participate and formally consent to participate.

The inclusion criteria for patients were: (1) aged 18 years and older; (2) met criteria for an SMI per self-report and confirmed by referring provider or administration of the Mini International Neuropsychiatric Interview (MINI; DSM-5, Version 7.0.0) by a licensed clinical social worker on the research team^3^; (3) exhibited a sleep or circadian disturbance as determined by endorsing 4 (quite a bit) or 5 (very much), or the equivalent for reverse scored items, on one or more PROMIS-Sleep Disturbance questions [47, 48]; (4) guaranteed place to sleep for at least two months that was not a shelter; (5) received the standard of care for the SMI and consent to regular communications between the research team and provider; and (6) consented to access their medical record and participate in assessments.

Patients were excluded if they met any of the following criteria: (1) presence of an active and progressive physical illness or neurological degenerative disease directly related to the onset and course of the sleep and circadian problems, or that made participation in the study unfeasible, as assessed by the Checklist of Medical Conditions and Symptoms on the Duke Structured Interview for Sleep Disorders [49] and clinical interview; (2) presence of substance abuse/dependence only if it made participation in the study unfeasible; (3) current active intent or plan to commit suicide (those with suicidal ideation were eligible) only if it made participation in the study unfeasible, or homicide risk; (4) night shift work for more than two nights per week in the past three months (i.e., regularly scheduled work from 12 a.m. – 6 a.m.); or (5) pregnant or breastfeeding.

### Facilitation

As described above as well as in the protocol paper [35] and in Additional File 1, facilitation was selected as the core implementation strategy based on promising evidence (e.g., [28, 50, 51]). Facilitation refers to the “multi-faceted interactive process of problem solving, enabling and supporting individuals, groups, and organizations in their efforts to adopt and incorporate innovations into routine practices” (p. 52). It is grounded in the integrated Promoting Action on Research Implementation in Health Services (i-PARIHS) framework [8]. In the present study, each CMHC received direct support from the lead facilitator, who is a licensed clinical social worker with expertise in community mental health and sleep treatment (ERA), and a team of trained facilitators employed by the research team. Throughout the study, the facilitation team was supervised by the Principal Investigator (PI; AGH) with periodic check-ins with a REP and facilitation expert (AMK). Facilitation activities were also informed by the Veterans Affairs facilitation manual [52] and Harvey and Kitson’s [53] Facilitation Guide. Additionally, the lead facilitator (ERA) and postdoctoral scholar (LDS) completed the Behavioral Health Veterans Affairs Quality Enhancement Research Initiative Implementation (BH QUERI) Facilitation Training and regularly attended BH QUERI’S monthly drop-in consultation group. Day-to-day facilitators conducted ongoing assessments at each CMHC site and planned responses to address unmet needs and reduce barriers via an integrated set of evidence-based implementation strategies [28, 29].

Given the i-PARIHS framework’s emphasis on facilitation as a “flexible and responsive way to tailor” the implementation process to a “particular issue, setting and people involved” (p. 6; 8), the facilitators submitted an Implementation Log weekly for 17 months, yielding almost 4,000 h of facilitation-related activities. The purpose of the Implementation Log was to provide theory-based tracking of facilitation activities in near ‘real time’. Knowing that the processes and strategies involved in implementation are complex and not always described in research reports, presenting barriers to replication and translatability, the log was designed to track a comprehensive range of key implementation variables. The log was developed following Proctor et al.’s [54] reporting guidelines for research on implementation strategies. All variables for the log were derived through piloting, collaboration with the facilitators, and/or based on standardized frameworks, taxonomies, and guidelines from the implementation science literature. As described in the main report of the Implementation Log data [55], the Log was formatted in Excel. Seven key variables from Proctor et al. [54] were tracked: action, actor, action target, temporality, dose, implementation strategies and intended outcomes [54]. Using Excel, facilitators completed a new log each day. Their daily log included the date (i.e., for temporality) and the “events,” or actions they completed that day. Each event was logged in a new row. For each event, facilitators entered 11 variables, presented in sequential columns as follows: County; Time; Communication Type; To Whom the Target Applies; Target; Implementation Strategy 1; Implementation Strategy 2; Implementation Strategy 3; Implementation Strategy 4; Intended Outcome; and To Whom the Outcome Applies. Lists of all variable options and most corresponding definitions were included in the log for facilitators to reference [54]. In addition, qualitative analyses using deductive and inductive coding were used to analyze data from a semi-structured interview that assessed facilitator perceptions of the log with respect to acceptability, appropriateness, and feasibility [54].

### Interventions

Two variations of TSC were tested: Standard TSC and AdaptedTSC. Both were delivered alongside the usual care offered by each CMHC. The control condition was UC-DT. In the CMHCs, usual care consisted of working with a service provider (e.g., psychologist, case manager, occupational therapist, psychiatrist, nurse practitioner) who provides direct mental health support alongside other services as needed (e.g., housing support).

Although most providers delivered TSC via individual sessions, some opted to deliver it in a group setting. Note that TSC was originally developed in English, then translated into Spanish to expand access. TSC was offered by 18 Spanish-speaking providers to 35 Spanish-speaking patients.

#### Standard TSC

Standard TSC was delivered by CMHC providers across eight 50-min, weekly sessions (Harvey & Buysee, 2017). It was comprised of 4 cross-cutting modules featured in every session, 4 core modules, and 7 optional modules used based on clinical presentation, treatment goals, and provider case conceptualization (see Additional File 1 for description). Training for the Standard TSC condition consisted of a 1-day workshop (i.e., 6–8 h) or two, 3-h training blocks, based on CMHC preference.

#### Adapted TSC

We grounded the process for adapting TSC in theory, data, and end-user input (see Additional File 1 for further details). Adapted TSC was delivered by CMHC staff across four, 20-min, weekly sessions (see Additional File 1 for description). Treatment consisted of the same four *cross-cutting modules* as in Standard TSC as well as three of the core modules and one of the optional modules. Training for the Adapted TSC condition consisted of four, 1-h workshops or two, 2-h workshops, based on CMHC preferences.

#### UC-DT

In UC-DT, patients began with usual care for four or eight weeks, depending on whether their CMHC was randomized to Adapted TSC or Standard TSC. After the delay, they received Adapted or Standard TSC, similarly based on the condition to which their CMHC had been randomized. The decision to include UC-DT as the comparison condition was made based on advice from the early CMHC partners to strike a balance between (a) including a comparison group to demonstrate the effectiveness of TSC in community settings; (b) ensuring that *all* participants receive what we hypothesize to be an active treatment (TSC); and (c) maximizing efficiency in terms of study duration, budget, and participants’ time investment. Notably, usual care has been the comparison group in several influential studies [42, 56].

## Measures

In addition to the measures below, a sociodemographics form was completed by providers and patients.

### Providers

#### Primary outcome

##### Acceptability

Providers rated the acceptability of TSC via the *Acceptability of Intervention Measure* (AIM) [57]. This 4-item measure was rated on a scale from 1 (completely disagree) to 5 (completely agree). This measure has demonstrated satisfactory validity, internal reliability, test–retest reliability, and sensitivity to change [57].

#### Secondary outcomes

##### Appropriateness and feasibility

Providers rated the appropriateness and feasibility of TSC via the following 4-item measures: *Feasibility of Intervention Measure* (FIM) and *Intervention Appropriateness Measure* (IAM) [57] using the same scale as the AIM.

#### Other measures

##### Number of TSC sessions

The number of sessions delivered to each enrolled patient by each provider was counted.

##### Occupation

Providers were asked to report their current position, professional degree, and work history, including their caseload, theoretical orientation, licensure status, and previous training in sleep treatment.

### Patients

#### Primary outcome

##### Sleep disturbance

The 8-item PROMIS-Sleep Disturbance (PROMIS-SD) assessed disruption to sleep (e.g., trouble staying asleep) over the past seven days. Items were rated on a scale from 1 (not at all/never/very poor) to 5 (very much/always/very good). T-scores were used (Yu et al., 2011), calculated by summing the raw scores and using conversion tables on healthmeasures.net, where higher scores indicate more severe symptoms. This measure has demonstrated acceptable reliability and validity [47, 48].

#### Secondary outcomes

##### Sleep-related impairment

The 8-item^4^ PROMIS-Sleep Related Impairment (PROMIS-SRI) assessed daytime impairment related to sleep problems using the same scale, timeframe an scoring as the PROMIS-SD.

##### Functional impairment

Functional impairment was assessed via the Sheehan Disability Scale (SDS) [58]. Impairment in work and school, social life, and home and family was rated via three items on a scale from 0 (not at all) to 10 (extremely). Scores ranged from 0–30, with higher scores indicating greater impairment. This measure demonstrated good reliability and validity [58].

##### Overall sleep health

The Sleep Health Composite captures overall sleep health for the complexity of sleep problems in SMI that are covered by TSC [59]. It is defined as the sum of scores on six sleep health dimensions (each dimension dichotomized as 1 = good; 0 = poor): Regularity (midpoint fluctuation), Timing (mean midpoint), Efficiency (sleep efficiency), Duration (total sleep time), Satisfaction (sleep quality question on PROMIS-SD), and Alertness (daytime sleepiness question on PROMIS-SRI). All dimensions – except Satisfaction and Alertness – were assessed via questions about sleep–wake patterns over the past seven days. Scores ranged from 0–6, with higher scores indicating better sleep health. Initial validity of this measure has been established [59].

##### Psychiatric symptoms

The DSM-5 Cross-Cutting Measure assessed psychiatric symptoms across 13 mental health domains. Participants rated how often they were bothered by each symptom on a scale from 0 (not at all) to 4 (nearly every day). Scores ranged from 0–52, with higher scores indicating more severe symptoms. This measure has demonstrated good test–retest reliability and clinical utility [60, 61].

#### Exploratory outcomes

##### PhenX toolkit

[62] (see Additional File 2 for further details). To assess suicidal ideation and behaviors, two subscales from the screening version of the Columbia-Suicide Severity Rating Scale—Severity of Suicidal Ideation and Suicidal Behavior—were used. Ideation was assessed in the past month and suicidal behavior in the past three months. These scales were scored according to the scoring guide [63]. For ideation, the highest numerical value (i.e., the value associated with the most severe item endorsed, ranging from 1 to 5) was used as the final score. For suicidal behavior, five suicide-related behaviors were assessed by separate items, scored with a binary scale (0 = no, 1 = yes) and frequency of patients who endorsed a given behavior was identified. The PhenX ‘Alcohol – 30-Day Quantity and Frequency’, ‘Tobacco – 30 Day Quantity and Frequency’, ‘Substances – 30-Day Frequency’, and ‘Supplemental Beverage Questionnaire’ were used to assess alcohol, tobacco, psychoactive substance, and caffeine consumption over the past 30 days.

##### Credibility and perceived improvement

Perceptions of TSC credibility and symptom improvement were assessed by four questions adapted from the Credibility/Expectancy Questionnaire (CEQ) (Devilly & Borkovec, 2000). These questions assessed (1) how logical TSC seemed, (2) how successful it was in reducing sleep symptoms, (3) how confident patients would be in recommending TSC to a friend, and (4) how much improvement patients believe had occurred. All questions were rated on a scale from 0 (not at all) to 9 (very), except for perceived improvement, which was rated as a percentage from 0–100%.

##### UC-DT contamination

At the end of UC-DT and before starting TSC, we assessed for patient exposure to TSC during the UC-DT waiting period by asking “Have you received any sleep intervention, treatment, or coaching since entering the study?” If yes, the assessor asked for details. Two independent coders rated the responses for potential exposure to TSC.

### Procedure

CMHCs and patients were randomized through a computerized randomization sequence. When randomizing patients, we stratified for presence of psychosis or not (current), presence of substance use or not (current) and age (≥ 50 or not), as there was evidence these variables can impact sleep or treatment outcome [18, 64, 65]. Only the facilitators, assessors, and research team (i.e., not CMHCs, providers, or patients) were privy to which CMHCs and patients were allocated to which TSC treatment condition (Adapted versus Standard TSC). CMHC providers and patients knew whether a patient had been randomized to receive the immediate or delayed treatment. Facilitation was selected as the core implementation strategy used to implement TSC, as described above. The launch of this study coincided with the COVID-19 pandemic. Thus, most study processes were conducted virtually. The methods described below have been published, often in more detail, in the protocol paper [35].

Providers and patients were consented by the assessment team prior to participation. All patients were compensated for their participation, and providers were compensated if permitted by their CMHC. The assessments were completed by the assessment team, comprised of experienced assessors. Because the assessors needed to provide study-related information—such as number of assessments and treatment sessions—to the patients during the consent process, the assessors were not blind to condition at pre-treatment. However, at post-treatment and the 6 FU, we endeavored to keep assessors blind to condition. Assessors received ongoing supervision and were thoroughly trained to deliver the surveys with integrity and minimal bias.

For Aim 1, the patient assessments for the immediate TSC condition were completed at pre-treatment and post-treatment. In UC-DT, the patient assessments for Aim 1 were completed at pre-treatment and four or eight weeks after pre-treatment (i.e., at the end of usual care and before delayed treatment with TSC), depending on whether their county had been randomized to Adapted or Standard TSC, respectively. We did collect a 6-month follow-up (6 FU) for immediate TSC but we did not include these in the analyses because the prespecified analyses for Aim 1 focus on timepoints with a comparable UC-DT comparison group. For Aim 2, provider assessments of acceptability, feasibility, and appropriateness were measured at post-training, mid-treatment, and post-treatment. Following the prespecified analyses, only the change from post-training to post-treatment was examined because change at post-treatment was the primary effect of interest, and we sought to minimize risks of additional comparisons. For Aim 3, an attempt was made to collect mid-treatment but this was largely not successful. Hence, mid-treatment data was not used for the mediation analysis. For Aim 3 and exploratory Aim 1, as delineated in the prespecified analyses, both post-treatment and 6 FU were included for immediate TSC to examine effects of TSC treatment condition (Standard vs. Adapted TSC) on patient outcomes, because (a) assessing change to post-treatment and 6 FU (i.e., immediate and sustained change) were both high priority and (b) comparable comparisons for Adapted and Standard TSC were available at both timepoints. For exploratory Aim 2, treatment effects were examined only from pre- to post-treatment, because as with Aim 1, comparable comparisons between TSC and UC-DT were only available at these timepoints.

Information on the recruitment of CMHCs, providers and patients is available in Additional File 2.

### Trial registration, data transparency and openness

All research materials, data, and analysis code are available from the authors upon request. This study was preregistered on clinicaltrials.gov (identifier: NCT04154631), a protocol paper was published [35] and the study received approval from the Committee for the Protection of Human Subjects at the University of California, Berkeley. Updates made to the clinicaltrials.gov protocol (identifier: NCT04154631) in December, 2022 are summarized in the protocol paper. Since then, in March 2023 we (a) clarified that the protocol covered the Implementation Phase and not the other phases, (b) we separated the entry for the Utilization Questionnaire into two entries, one for post-treatment and the other for 6 FU and (c) we clarified the timeframes for several measures.

### Power analysis

A pre-specified power analysis was conducted for the entire trial, which included providers and patients from the implementation phase (i.e., CMHC providers trained by the UC Berkeley team and the focus of the present study) [35] and the train-the-trainer phase (i.e., CMHC providers who were trained by local trainers, who had been trained by the UC Berkeley team) [45]. However, these two phases were subsequently separated to more thoroughly investigate results from each phase of the study (i.e., implementation phase and train-the-trainer phase). Thus, for the present study, the minimum detectable effect sizes (MDES) were calculated with the sample from the implementation phase for the primary outcomes: sleep disturbance for patients and acceptability for providers. Optimal Design software for cluster-randomized trials with repeated measurements was used to calculate the MDES [66, 67]. The intraclass correlation coefficients (ICC) were calculated for multilevel models with timepoints (level 1) nested within patients or providers (level 2) nested with CMHCs (level 3). The resulting ICCs were very small (< 0.0001). To be conservative, an ICC of 0.001 was used. For sleep disturbance, using this ICC of 0.001, the final sample size of *N* = 396 patients, alpha of 0.05, 10 CMHCs, and power = 0.80, the MDES was 0.42. Based on the effect sizes yielded for the main patient-level analyses, this MDES was exceeded. For acceptability, using this ICC, the final sample size of *N* = 93 providers, alpha of 0.05, 10 CMHCs, and power = 0.80, the MDES was 0.96. To account for the possibility that the provider analyses may have been underpowered, significance values and effect sizes are emphasized for providers.

### Analysis plan

Analyses generally followed the plan specified in the protocol paper [35]. Deviations from this plan have been noted in the sections below. Information about assumption checks, missing data and covariates are included in the Additional File 3 and Additional File 4, Tables 1, 2 and 3.

#### Analyses

All analyses were conducted with Stata Version 16.1.

##### Multilevel Models (Aims 1 & 2 and Exploratory Aims 1 & 2)

For Aims 1 & 2 and Exploratory Aims 1 & 2, multilevel models (MLMs) were used to account for multiple observations nested within patient [68]. Analyses used intent-to-treat principles and maximum likelihood estimation, which performs well in simulations of MLMs with missing data up to 50% [69]. All models used robust standard errors. Effect sizes for all multilevel models are represented with ‘*d*’ and were calculated following Feingold ([70], Eq. 5), using unadjusted change scores and raw standard deviations at pre-treatment from each treatment condition. The Benjamini–Hochberg procedure [71] was used to correct for multiple testing on the primary outcomes (sleep disruption and acceptability), per the protocol paper. All MLMs compared pre-treatment to post-treatment and, for level 1, included a dummy-coded time indicator as the predictor (1 = post-treatment, pre-treatment as the reference). Exploratory Aim 1 also compared pre-treatment to six-month follow-up and included an additional time indicator accordingly. The level 2 equation included dummy-coded treatment condition (*Aim 1 and Exploratory Aim 2:* 1 = immediate TSC, with UC-DT as the reference; *Aim 2 and Exploratory Aim 1:* 1 = Adapted TSC, with Standard as reference) and treatment-by-time interaction terms, which were the parameters of interest. Additionally, Exploratory Aim 2 included three-way interactions between time, treatment, and the following pre-specified moderators: sex (dummy coded: 0 = male, 1 = female), age (dummy coded: 0 = < 50, 1 = ≥ 50, and continuous baseline variables of PROMIS-SD, PROMIS-SRI, SDS, and DSM-5 Cross-Cutting. As part of the prespecified analyses for Exploratory Aim 1, linear regression models were also used to test the effects of TSC treatment condition on credibility and perceived improvement at post-treatment. The predictor was dummy-coded TSC treatment condition (1 = Adapted TSC, with Standard as reference) and the outcomes were credibility, expectancy, and total CEQ. Effect sizes for linear regressions are partial eta squared, or the proportion of variance explained by the predictor of interest [72].

Almost all outcomes were continuous, except for the following binary outcomes tested in Exploratory Aim 1: suicidal thoughts and behaviors and illicit substance use. For these outcomes, multilevel logistic regression was used. However, because few participants endorsed these items, the models would not converge. Instead, the frequencies of patients’ endorsement of each item are presented in Additional File 4, Table 4.

We list the outcomes included in each MLM (see Additional File 3 for covariates and below for SEMs). For the Aim 1 MLMs, the outcomes were PROMIS-SD, PROMIS-SRI, Sleep Health Composite, DSM-5 Cross-Cutting, and SDS. Note that, in the protocol paper, the Sleep Health Composite was listed in the Measures section but was omitted from the planned analysis section in error. For Aim 2, the outcomes were providers’ perceptions of acceptability, feasibility, and appropriateness. For Exploratory Aim 1, the MLM outcomes were severity of suicidal ideation, average cigarettes per day among people who endorsed using tobacco, average number of caffeinated drinks per day, and number of days the patient consumed alcohol in the past 30 days. As noted above, the linear regression outcomes Exploratory Aim 1 were credibility, expectancy, and total CEQ. For Exploratory Aim 2, the outcomes mirrored Aim 1 and were PROMIS-SD, PROMIS-SRI, Sleep Health Composite, DSM-5 Cross-Cutting, and SDS.

##### Structural Equation Modeling (SEM) (Aims 1 & 3)

For the mediation models in Aims 1 and 3, SEM was used. Specifically, the analysis of covariance (ANCOVA) approach was used, in which pre-treatment measures of the mediator and outcome are included as covariates.^5^ This approach has been recommended for designs comparing pre- to post-treatment [73]. In particular, statisticians and methodologists have argued that contemporaneous models, whereby the mediators and outcomes are both measured at post-treatment, may confer advantages for clinical trials, because these timepoints capture the interval during which the greatest changes are most likely to occur in the mediators and outcomes (e.g., [73–75]). For Aim 1, the predictor was condition (immediate TSC vs. UC-DT), the mediator was PROMIS-SD or PROMIS-SRI at post-treatment, and the outcomes were DSM-5 Cross-Cutting and SDS at post-treatment. For Aim 3, the predictor was TSC condition (Adapted vs. Standard), the mediator was AIM, FIM, or IAM at post-treatment, and the outcomes were PROMIS-SD, PROMIS-SRI, DSM-5 Cross-Cutting, and SDS at post-treatment and six-month follow-up. For all SEMs, the parameter of interest was the indirect effect. Maximum likelihood estimation was used. As noted above, all models were run with robust standard errors. Effect sizes for mediation models are the mediated proportions (MP), or the proportion of the total effect that is explained by the indirect effect expressed as a percentage [76].

## Results

**Fig. 1:**
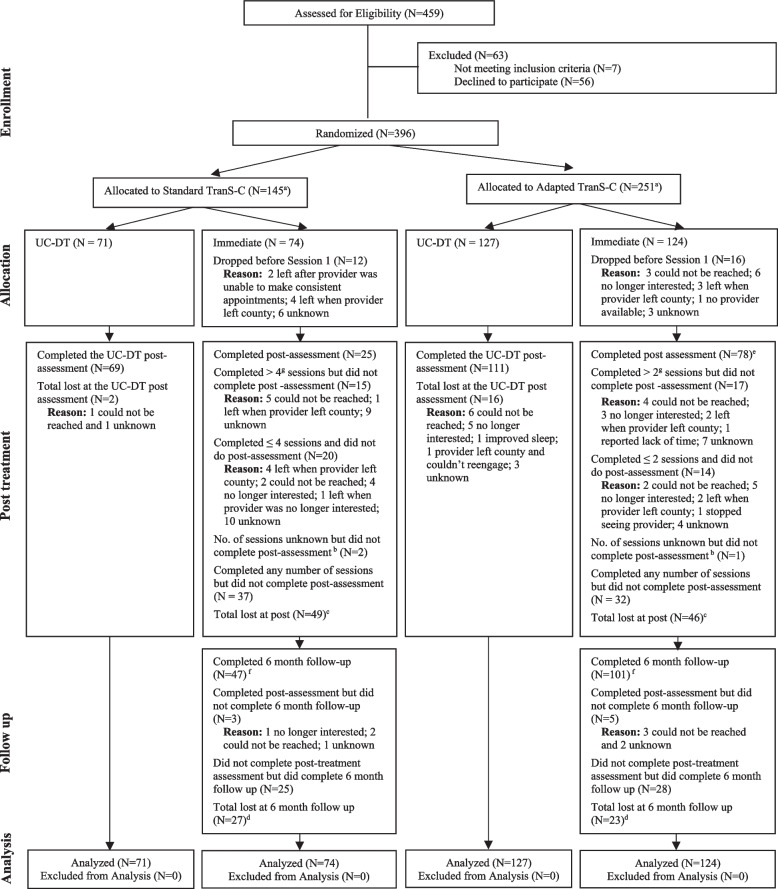
CONSORT Diagram Illustrating the Flow of Patients Through the Study Note. ^a^The larger N in Adapted vs. Standard TranS-C was a result of stronger recruitment in the counties randomized to this condition. ^b^We could not determine the count of sessions completed when we when we lost contact with provider and client. ^c^Total lost at post is calculated by summing those who dropped before session 1 and those who completed any number of sessions but did not complete a post assessment. In the immediate condition the 2 participants who completed a post-assessment who dropped before session 1 are subtracted from this sum. ^d^Total lost at 6-month follow up is calculated by subtracting those who completed follow-up from the initial N. ^e^Out of 78 who completed post-assessment, 2 dropped before Session 1. ^f^6-month follow-up was 6 months from the end of treatment. ^g^Drop out is defined as completing half of the number of sessions which is 2 for Adapted and 4 for Standard

**Fig. 2:**
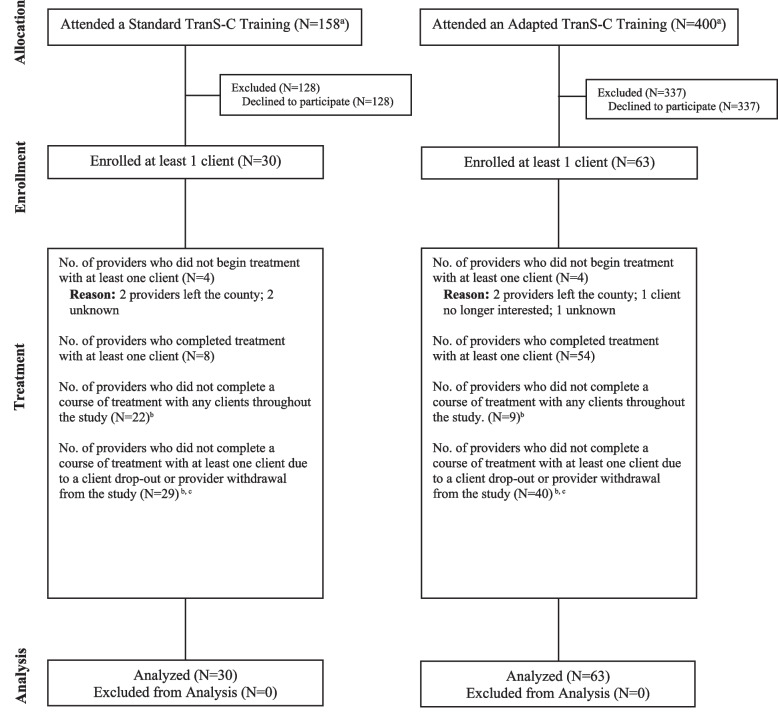
CONSORT Diagram Illustrating the Flow of Providers Through the Study Note. ^a^The larger N in Adapted vs. Standard TranS-C was a result of stronger recruitment in the counties randomized to this condition. ^b^As providers often treated multiple clients, the reason for non-completion varied based on the client or the timing of the provider’s departure from the county. Reasons for providers not completing a course of treatment included the provider leaving the county or study, clients no longer interested, and unknown factors. (See Fig. 1 for more details about treatment dropouts). ^c^As providers were often matched with more than one client, the categories listed may overlap and thus do not sum to the total number of enrolled providers

See Fig. 1 for the CONSORT diagram for patients which includes, per the protocol paper, dropout rates at different stages of the trial. Dropout rates for participants in the immediate TSC condition were 14.14% (28 participants) before session 1, 34.48% (69 participants) between session 1 and the post-treatment assessment, and 4.04% (8 participants) between post and 6 FU. Attrition rates were significantly higher in Standard than Adapted TSC during the treatment phase (50% in Standard; 25.81% in Adapted; χ^2^ = 11.00, df = 1, *p* < 0.001), but not significantly different prior to Session 1 (16.22% in Standard; 12.90% in Adapted; χ^2^ = 0.19, df = 1, *p* = 0.70), or at 6 FU (4.05% in Standard; 4.03% in Adapted; χ^2^ < 0.01, df = 1, *p* = 1.00). Relative to completers, participants who did not begin treatment or who dropped out were not significantly different on stratification factors: sex (χ^2^ = 1.30, df = 1, *p* = 0.30), age group (above or below 50 years; χ^2^ = 0.72, df = 1, *p* = 0.40) or psychosis status (χ^2^ = 0.06, df = 1, *p* = 0.80). See Fig. 2 for the CONSORT diagram for providers.

As evident in Table 2, Standard and Adapted TSC did not differ on any pre-treatment patient demographic variable except on government assistance (*p* = 0.05) and education (*p* = 0.07), both of which approached significance. Specifically, more patients in Adapted had completed or had some graduate school education (8.06%) than in Standard (1.35%). Additionally, more patients in Standard reported using Medicaid (37.84%) than in Adapted (23.39%), while a greater number in Adapted were receiving Supplemental Social Security Income/Social Security Disability Insurance (SSI/SSDI) (25.81%) than in Standard (12.16%). As evident in Table 3, there were no significant differences in demographics between providers in Standard versus Adapted TSC. Additional File 4, Table 5 presents the patient demographics by treatment condition and immediate TSC vs. UC-DT which did not differ on any pre-treatment patient demographic variable except education (*p* = 0.07) approached significance.

**Table 2:** Patient Demographics and Number of Sessions by Treatment Condition (Standard versus Adapted TSC) at Pre-Treatment

Characteristic	Standard TSC (*n* = 74)		Adapted TSC (*n* = 124)		χ^2^	*p*-value
	*n*	%	*n*	%		
Sex					3.43	0.18
Female	42	56.76	83	66.94		
Male	31	41.89	41	33.06		
Missing/declined to answer	1	1.35	0	0.00		
Ethnicity					0.79	0.67
Hispanic or Latino	22	29.73	44	35.48		
Not Hispanic or Latino	51	68.92	79	63.71		
Missing/declined to answer	1	1.35	1	0.81		
Race					3.97	0.78
American Indian/Alaska Native	9	12.16	13	10.48		
Native Hawaiian/Pacific Islander	3	4.05	2	1.61		
Asian	7	9.46	7	5.65		
Black or African American	5	6.76	13	10.48		
White	40	54.05	68	54.84		
More than one race	4	5.41	6	4.84		
Other/category not listed	4	5.41	12	9.68		
Missing/declined to answer	2	2.70	3	2.42		
Education					8.50	0.07
High school graduate or below	21	28.38	40	32.26		
Some or completed college or vocational school	50	67.57	73	58.87		
Some or completed graduate school	1	1.35	10	8.06		
Other/category not listed	0	0.00	1	0.81		
Missing/declined to answer	2	2.70	0	0.00		
Employment					4.12	0.39
Full-time	11	14.86	16	12.90		
Part-time	12	16.22	26	20.97		
Not employed	46	62.16	78	62.90		
Other/category not listed	3	4.05	4	3.23		
Missing/declined to answer	2	2.70	0	0.00		
Civil Status					3.45	0.18
Partnered	12	16.22	19	15.32		
Unpartnered	60	81.08	105	84.68		
Missing/declined to answer	2	2.70	0	0.00		
Living Arrangement					7.41	0.19
Alone	9	12.16	31	25.00		
With family	49	66.22	66	53.23		
With friend or roommate or pet	11	14.86	17	13.71		
Supportive housing	2	2.70	6	4.84		
Other/category not listed	2	2.70	4	3.23		
Missing/declined to answer	1	1.35	0	0.00		
Government Assistance^a^					17.10	0.05
Unemployment	6	8.11	4	3.23		
Medicare	7	9.46	22	17.74		
Medicaid	28	37.84	29	23.39		
Social Security	8	10.81	14	11.29		
Food Stamps	25	33.78	34	27.42		
SSI/SSDI	9	12.16	32	25.81		
SNAP	2	2.70	4	3.23		
None	0	0.00	4	3.23		
Other/category not listed	7	9.46	21	16.94		
Missing/declined to answer	20	27.03	29	23.39		
Annual Personal Income					6.84	0.45
< $10,000	18	24.32	39	31.45		
$10,000-$20,000	21	28.38	36	29.03		
$20,000-$30,000	8	10.81	12	9.68		
$30,00-$40,000	4	5.41	4	3.23		
$40,000-$50,000	1	1.35	5	4.03		
> = $50,000	2	2.70	7	5.65		
I don’t know my income	19	25.68	21	16.94		
Missing/declined to answer	1	1.35	0	0.00		
Annual Household income					6.80	0.45
< $10,000	10	13.51	23	18.55		
$10,000-$20,000	16	21.62	31	25.00		
$20,000-$30,000	11	14.86	10	8.06		
$30,00-$40,000	4	5.41	6	4.84		
$40,000-$50,000	2	2.70	6	4.84		
> = $50,000	6	8.11	18	14.52		
I don’t know my income	24	32.43	28	22.58		
Missing/declined to answer	1	1.35	2	1.61		
Self-reported diagnosis^b^					9.62	0.65
Neurodevelopmental disorders	8	10.81	9	7.26		
Psychosis	23	31.08	40	32.26		
Bipolar Disorder	22	29.73	29	23.39		
Major Depressive Disorder	43	58.11	54	43.55		
Anxiety disorders	38	51.35	73	58.87		
Obsessive–compulsive and related disorders	2	2.70	6	4.84		
Trauma and stressor-related disorders	24	32.43	35	28.23		
Dissociative disorders	1	1.35	3	2.42		
Personality disorders	3	4.05	3	2.42		
Feeding and eating disorders	2	2.70	2	1.61		
Substance-related and addictive disorders	2	2.70	1	0.81		
Other/category not listed	1	1.35	3	2.42		
Missing/declined to answer	1	1.35	9	7.26		
	Mean	SD	Mean	SD	t	*p*-value
Age	38.84	13.56	42.42	16.36	−1.65	0.10
Education (years)	13.59	2.87	13.87	3.35	−0.61	0.54
No. of sessions received (all)^c^	5.43	6.76	3.85	2.71	1.93	0.06
No. of sessions received (completers)^d^	8.95	8.01	4.99	1.91	2.97	< 0.01

Chi-squared was used for categorical variables, and *t* tests were used for continuous variables

^a^Some patients endorsed more than one government assistance category

^b^Comorbidity was common

^c^Number of TSC sessions received by all enrolled patients in the study

^d^Number of TSC sessions received by patients who completed treatment

**Table 3:** Provider Demographics by TSC Treatment Condition (Standard versus Adapted TSC) at Pre-Treatment

**Characteristic**	Standard TSC (*n* = 30)		Adapted TSC (*n* = 63)		**χ**^**2**^	*p*-value
	*n*	***%***	*n*	***%***		
Sex					3.26	0.35
Female	23	76.67	47	74.60		
Male	5	16.67	5	7.94		
Other/category not listed	0	0.00	1	1.59		
Missing/declined to answer	2	6.67	10	15.87		
Ethnicity					0.09	0.95
Hispanic or Latino	9	30.00	17	26.98		
Not Hispanic or Latino	15	50.00	33	52.38		
Missing/declined to answer	6	20.00	13	20.63		
Race					5.11	0.53
American Indian/Alaska Native	0	0.00	2	3.17		
Asian	3	10.00	7	11.11		
Native Hawaiian or Pacific Islander	0	0.00	2	3.17		
Black or African American	2	6.67	1	1.59		
White	19	63.33	32	50.79		
More than one race	1	3.33	5	7.94		
Missing/declined to answer	5	16.67	14	22.22		
Degree Type					10.08	0.18
Marriage and Family Therapy	10	33.33	13	20.63		
Psychology	3	10.00	7	11.11		
Social Work	8	26.67	20	31.75		
Nursing	0	0.00	1	1.59		
Medical	1	3.33	0	0.00		
Occupational Therapy	0	0.00	5	7.94		
Other/category not listed	7	23.33	5	7.94		
Missing	4	13.33	12	19.05		
Therapeutic Approach^a^					9.45	0.22
Client Centered	19	63.33	40	63.49		
Family Systems	5	16.67	15	23.81		
CBT	21	70.00	30	47.62		
Psychodynamic	7	23.33	11	17.46		
Humanistic	2	6.67	0	0.00		
Integrative/Holistic	1	3.33	3	4.76		
None	0	0.00	3	4.76		
Missing/declined to answer	3	10.00	13	20.63		
Licensure					0.92	0.63
Licensed	16	53.33	32	50.79		
Not Licensed	11	36.67	20	31.75		
Missing/declined to answer	3	10.00	11	17.46		
	Mean	SD	Mean	SD	t	*p*-value
Age	38.26	10.06	41.54	11.38	0.97	0.34
Caseload	40.09	23.97	29.59	31.62	1.47	0.15
Employment Duration	3.87	3.51	3.84	3.96	0.03	0.98
Years Since Degree Earned	9.44	7.66	9.62	7.40	−0.09	0.93

^a^Some providers endorsed more than one therapeutic approach. Chi-squared was used for categorical variables, and *t* tests were used for continuous variables. *CBT* Cognitive behavioral therapy, *Caseload* Number of clients on caseload. Employment duration = length of time employed at current CMHC in years

Only four participants (2.02%) reported having potentially received a part of TSC during the UC-DT waiting period (e.g., “provider gave suggestions to alleviate anxiety before bed”, “completed one module”, “sleep diary with sleep therapist”). We deemed this to indicate minimal contamination.

### Aim 1

See Tables 4 and 5. TSC, relative to UC-DT, was significantly associated with improvements from pre- to post-treatment in sleep disturbance, sleep-related impairment, sleep health composite, psychiatric symptoms, and overall functional impairment. Sleep disturbance (primary outcome) withstood the Benjamini–Hochberg correction.

**Table 4:** Means, Standard Deviations, and Effect Sizes for Primary and Secondary Outcomes

	Pre-Treatment				Post-Treatment				
Patient Outcomes									
	UC-DT		TSC		UC-DT		TSC		d
	Mean	SD	Mean	SD	Mean	SD	Mean	SD	
PROMIS-SD*	62.80	7.89	62.76	7.15	61.65	8.33	50.83	10.07	−1.52
PROMIS-SRI	62.01	8.07	62.06	8.82	61.17	8.42	51.78	10.38	−1.06
SHC	2.07	1.33	1.99	1.46	2.22	1.49	3.54	1.55	0.95
DSM-5	24.18	9.43	24.3	8.97	23.35	8.82	18.88	10.62	−0.52
SDS	13.15	6.72	12.63	7.38	12.36	7.19	6.49	6.06	−0.71
Provider Outcomes									
	Standard TSC		Adapted TSC		Standard TSC		Adapted TSC		d
	Mean	SD	Mean	SD	Mean	SD	Mean	SD	
AIM*	4.69	0.41	4.70	0.46	4.64	0.63	4.67	0.53	0.06
FIM	4.62	0.46	4.61	0.48	4.61	0.74	4.61	0.52	0.02
IAM	4.62	0.5	4.69	0.46	4.59	0.69	4.59	0.54	−0.16

*PROMIS-SD* PROMIS Sleep Disruption, *PROMIS-SD* PROMIS Sleep Disturbance, *PROMIS-SRI* PROMIS Sleep-Related Impairment, *SHC* Sleep Health Composite (note, scored such that higher scores indicate better sleep health), *DSM-5* DSM-5 Cross-Cutting, *SDS* Sheehan Disability Scale, *AIM* Acceptability of Intervention Measure, *FIM* Feasibility of Intervention Measure, *IAM* Intervention Appropriateness measure, *TSC* Transdiagnostic Intervention for Sleep and Circadian Dysfunction, *UC-DT* Usual care followed by delayed treatment with TSC

*indicates primary outcome. Effect sizes are represented with ‘*d*’ and were calculated following Feingold (2009, Eq. 5), using unadjusted change scores and raw standard deviations at pre-treatment from each treatment condition

**Table 5:** Aim 1: Multilevel Modeling Results for Treatment Condition (UC-DT versus TSC) on Patient Outcomes from Pre- to Post-Treatment

	***b***	**SE**	*p*-value
**Outcome**			
PROMIS-SD	−10.91	1.94	** < 0.001**
PROMIS-SRI	−9.52	1.95	** < 0.001**
SHC	1.63	0.35	** < 0.001**
DSM-5	−6.72	1.46	** < 0.001**
SDS	−5.12	1.34	** < 0.001**

Bold indicates significant *p*-values. *b* = time-by-treatment interaction.

*SE* Robust standard errors, *PROMIS-SD* PROMIS Sleep Disturbance, *PROMIS-SRI* PROMIS Sleep-Related Impairment, *SHC* Sleep Health Composite, *DSM-5* DSM-5 Cross-Cutting, *SDS* Sheehan Disability Scale

See Table 6 for Aim 1 SEM results. The indirect effect of sleep disturbance on the relations between treatment condition (TSC vs. UC-DT) and psychiatric symptoms approached significance. When county was included as covariate, the indirect effect of sleep disturbance on the relations between treatment condition (TSC vs. UC-DT) and psychiatric symptoms was significant (−2.49, 95% CI [−4.56, 0.43], MP = 36.44%). The indirect effect of sleep disturbance on the relations between treatment condition on overall functional impairment was significant. Similarly, the indirect effects of sleep-related impairment on the relations between treatment condition (TSC vs. UC-DT) and psychiatric symptoms and overall functional impairment were significant. The indirect effects explained 27.76% to 70.30% of the total effects in these models.

**Table 6:** Aim 1: Mediation Models of Sleep Outcomes on Relations between Treatment Condition (TSC vs. UC-DT) and Psychiatric Symptoms and Overall Functional Impairment at Post-Treatment

	coefficient	SE	z	*p*	95% Confidence Interval of effect	%MP
**Aim 1 Model 1: TSC vs. UC-DT PROMIS-SD DSM-5 POST**						
Path a	−12.40	1.66	−7.48	< 0.001	−15.65, −9.15	-
Path b	0.15	0.08	1.88	0.06	−0.01, 0.31	-
Total effect	−6.70	1.26	−5.31	< 0.001	−9.17, −4.23	-
Indirect effect	−1.86	1.05	−1.76	0.08	−3.93 −0.21	27.76%
**Aim 1 Model 2: TSC vs. UC-DT PROMIS-SD SDS Post**						
Path a	−12.38	1.66	−7.44	< 0.001	−15.64, −9.12	-
Path b	0.25	0.06	4.45	< 0.001	0.14, 0.36	-
Total effect	−5.27	1.17	−4.51	< 0.001	−7.56, −2.98	-
Indirect effect	−3.12	0.74	−4.20	** < 0.001**	**−4.58, −1.66**	59.20%
**Aim 1 Model 3: TSC vs. UC-DT PROMIS-SRI DSM-5 POST**						
Path a	−10.59	1.79	−5.92	< 0.001	−14.09, −7.08	-
Path b	0.24	0.08	2.95	0.003	0.08, 0.39	-
Total effect	−6.94	1.26	−5.49	< 0.001	−9.41, −4.46	-
Indirect effect	−2.51	1.06	−2.37	**0.02**	**−4.58, −0.44**	36.17%
**Aim 1 Model 4: TSC vs. UC-DT PROMIS-SRI SDS POST**						
Path a	−10.60	1.81	−5.85	0.001	−14.14, −7.05	-
Path b	0.36	0.05	7.42	< 0.001	0.26, 0.46	-
Total effect	−5.42	1.17	−4.62	< 0.001	−7.72, −3.12	-
Indirect effect	−3.81	0.81	−4.69	** < 0.001**	**−5.41, −2.22**	70.30%

Significant effects for parameters of primary interest (i.e., indirect effects) are bolded

"-"indicates that value is not relevant to model

SE = robust standard errors

%*MP* Mediated proportion (i.e., the proportion of the total effect that is explained by the indirect effect expressed as a percentage)

Path a = path from the independent variable to mediator (i.e., Treatment condition PROMIS-SD or PROMIS-SRI)

Path b = path from the mediator to the outcome (PROMIS-SD or PROMIS-SRI DSM-5 Cross-Cutting or SDS)

All models adjusted for pre-treatment levels of the relevant mediator (i.e., PROMIS-SD or PROMIS-SRI) and relevant outcome (i.e., DSM-5 Cross-Cutting or SDS)

*TSC* Transdiagnostic Intervention for Sleep and Circadian Dysfunction, *UC-DT* Usual care followed by delayed treatment with TSC, *PROMIS-SD* PROMIS Sleep Disturbance, *PROMIS-SRI* PROMIS Sleep-Related Impairment, *SDS* Sheehan Disability Scale, *DSM-5* DSM-5 Cross-Cutting, *POST* Post-treatment assessment

### Aim 2

See Table 7 for Aim 2 MLM results. TSC condition (Standard versus Adapted) was not significantly associated with providers’ perceptions of acceptability, feasibility, or appropriateness at post-treatment, accounting for pre-treatment.

**Table 7:** Aim 2: Multilevel Modeling Results for TSC Condition (Adapted vs. Standard) on Provider Perceptions of Treatment Fit from Pre- to Post-Treatment

	***b***	**SE**	*p* value
Outcome			
AIM	−0.03	0.11	0.77
FIM	−0.01	0.14	0.94
IAM	−0.11	0.12	0.34

*b* = time-by-treatment interaction

*SE *Robust standard errors*, AIM *Acceptability of Intervention Measure*, FIM *Feasibility of Intervention Measure*, IAM *Intervention Appropriateness measure

### Aim 3

See Additional File 4, Table 6 for path coefficients and standard errors of Aim 3 SEMs. There were no significant indirect effects of acceptability on the relations between TSC condition (Adapted versus Standard) and the primary or secondary patient outcomes of sleep disturbance, sleep-related impairment, psychiatric symptoms, or overall functional impairment. The indirect effects explained 3.13% or less of the total effects for each of these models. This pattern of results held for appropriateness and feasibility, such that the indirect effects explained 15.23% or 11.16% or less of the total effects, respectively.

### Exploratory aims

See Additional File 4, Table 7 for means and effect sizes of exploratory outcomes by timepoint and treatment condition and Additional File 4, Table 8 for MLM results.

For Exploratory Aim 1, Adapted TSC, relative to Standard TSC, was associated with a significant decrease in number of cigarettes per day from pre-treatment to six-month follow-up (*b* = −7.57, *p* = 0.005, *d* = −0.58). There were no significant differences in suicidal ideation severity, average daily caffeine use, or past 30-day alcohol use (all *p*s > 0.10).

There were no differences between Adapted versus Standard TSC on credibility (*b* = 0.14, SE = 0.32, *p* = 0.67, n_p_^2^ = 0.01), perceived improvement (*b* = 6.78, SE = 9.09, *p* = 0.46, n_p_^2^ = 0.02, or total CEQ (*b* = 0.10, SE = 0.18, *p* = 0.59, n_p_^2^ = 0.01). Looking across conditions, at post-treatment, the mean of the credibility items was 7.80 (*SD* = 1.52) and mean perceived improvement was 64.0% (*SD* = 28.57).

For Exploratory Aim 2, see Additional File 4, Table 9 for the MLM results. None of the planned demographics (sex, age) or baseline clinical symptoms (sleep disruption, sleep-related impairment, psychiatric symptoms) moderated the effects of treatment (UC-DT vs. TSC) on any of the primary or secondary patient outcomes (i.e., sleep disruption, sleep-related impairment, overall sleep health, psychiatric symptoms, overall functional impairment) from pre- to post-treatment (all *p*s > 0.10).

## Discussion

The goal of this study was to determine if the use of theory, data and end-user perspectives to guide an adaptation of the Transdiagnostic Intervention for Sleep and Circadian Dysfunction (TSC) yielded better outcomes and improved the “fit” of TSC to community mental health centers (CMHCs), relative to the standard version. Table 1 and the discussion below interprets the findings of this implementation effort in the context of the i-PARIHS framework which defines successful implementation of an evidence-based practice or treatment (EBPT) as dependent on four key factors: the strength of the evidence supporting the innovation, the individuals or groups receiving it, the specific characteristics of the setting where it will be implemented, and the method used to facilitate its integration into that context.

We first focus on outcomes for the patient-level recipients of the innovation in the CMHC context. Consistent with the first hypothesis, relative to usual care and before delayed treatment, TSC (combining Adapted and Standard) was associated with reduced sleep disturbance, reduced sleep-related impairment as well as improved sleep-health, psychiatric symptoms and functional impairment at the post-treatment assessment. These findings align with prior research in which TSC has been administered for a range of different populations and non-CMHC settings (e.g., [77–79]). Additionally, while these findings replicate a prior study conducted in CMHCs with SMI patients [15], they extend knowledge in two key areas. First, in the prior study, the providers were employed, trained and supervised within the university setting. In the present study, the providers were CMHC employees. Thus, the present study demonstrates that CMHC providers deliver TSC with positive outcomes, above and beyond standard care, despite carrying heavy and complex caseloads and working within resource constrained CMHCs. As such, and following the NIH Stage Model [21], this study moves knowledge on TSC to Stage 3 by demonstrating “Efficacy in the Real World.” It is notable that a small group of patients in UC-DT (n = 4) were exposed to TSC during the waiting period, yet TSC was still more effective than UC-DT. Second, these results add to the existing evidence that a transdiagnostic treatment, designed to address a range of sleep and circadian problems experienced by a mixed diagnosis SMI sample, is helpful to patients seeking treatment in CMHCs [15]. Indeed, Table 1 describes the broad range of diagnoses participants reported, with anxiety disorders, trauma-related disorders, mood disorders and schizophrenia spectrum disorders being most common. Moreover, extending prior research showing that sleep treatment improves symptoms of comorbid mental health conditions (e.g., [14, 16, 80]), TSC’s benefits for functional impairment and psychiatric symptoms were mediated by improvements in sleep and circadian problems. Re-stating this result in terms of the Experimental Therapeutics Approach [81, 82], we found evidence that TSC engaged the intended target or mechanism of change—*sleep and circadian processes—*which in turn, predicted change in patient outcomes. Importantly, returning to the i-PARIHS framework, the patient-level results suggest that implementation was quite successful for the first group of recipients in this study; namely, people diagnosed with SMI. In other words, as theorized by i-PARIHS, the use of facilitation was effective in supporting providers to deliver the innovation (TSC) within the context of CMHCs, resulting in successful target engagement and improved outcomes for patients (recipients).

We next focus on findings for provider-level recipients of the innovation (TSC) in the CMHC context. The second hypothesis was that Adapted TSC, because it was adapted to improve fit to the context, would be rated by CMHC providers as better fitted to the CMHC context relative to Standard TSC. Contrary to the hypothesis, there were no significant differences between the treatment conditions on the provider ratings of fit, including providers’ perception of acceptability, feasibility and appropriateness. In other words, the adaptation process we undertook to improve fit of the innovation for recipients, that was guided by REP, involving theory, data and end-user perspectives—resulted in a treatment that is shorter and simpler (four × 20-min sessions) than the original version (eight × 50-min sessions), while still attracting positive ratings from the providers. This adds to the growing evidence for REP as a framework that prepares effective health interventions for implementation in routine practice settings [36]. Phases 1 (Pre-conditions) and 2 (Pre-implementation) of the REP framework were completed in preparation for the present study. The current study addresses Phase 3 (Implementation) of REP. Subsequent reports from other parts of this study will address Phase 4 (Maintenance and Evolution) of REP [45, 46].

The finding that Adapted and Standard TSC were both acceptable to providers might be explained by the relative advantages of each approach – a key characteristic of the innovation to be considered within the i-PARIHS framework (Harvey & Kitson, 2016). Specifically, Adapted TSC has the advantage that it is shorter and simpler and more easily delivered alongside the other interventions CMHC providers must deliver (e.g., housing support). Meanwhile, Standard TSC has the advantage that it more comprehensively treats a range of common sleep and circadian disorders. Interestingly, the lack of difference between the treatment conditions on provider ratings of fit suggests that the potential barriers to Standard TSC that we anticipated, such as time requirements and the complexity of the intervention, can be effectively managed in “real-life” routine practice settings. Given that many EBPTs follow a similar format to Standard TSC (i.e., multiple 50-min sessions), this finding bodes well for the feasibility of scaling other EBPTs to settings akin to CMHCs. On the one hand, given the high provider ratings for both Adapted and Standard TSC, either form may be useful, with the specific choice depending on the characteristics of the setting and the patients. On the other hand, in a separate qualitative study involving interviews with a subset of providers from the current study, providers expressed positive feedback for both versions of the treatment. However, for many outcomes, they showed a preference for Adapted TSC, while highlighting more unfavorable aspects of Standard TSC [83]. The latter finding underscores the value of multi-method approaches to gain insights from a variety of perspectives across a range of measures.

Finally, consistent with i-PARIHS, the Aim 2 provider-level results suggest that the use of facilitation was effective in supporting this second group of recipients in the study—CMHC providers—to deliver the innovation (TSC) despite the many challenges faced at the local, organizational and outer context of CMHCs. Facilitation at the provider-level is described in Additional File 1 and included a focus on goal setting, consensus building, audit and feedback and team building. More specifically, less popular offerings by the facilitation team included weekly drop-in supervision and as-needed consultation. More popular were presentations on advanced topics related to sleep and mental health (e.g., Lunch & Learn, Coffee Colloquium), setting up Continuing Education credits and working toward sleep treatment certification that CMHC providers could achieve via three supervised TSC cases. More formally, preliminary evidence from the facilitation log that was collected throughout the study (described earlier) indicated that facilitators spent most of their time on internal team meetings for ongoing problem solving and sharing updates; simplifying and clarifying communication with CMHC providers about research-related logistics and study administration; preparing training materials, including making training dynamic; and delivering trainings and workshops [55].

Not surprisingly given the results just discussed and contrary to the third hypothesis, there were no significant effects for Adapted TSC on the primary and secondary patient outcomes indirectly via higher provider ratings of acceptability, feasibility and appropriateness, relative to Standard TSC. Although the hypothesized indirect effects were not significant, the mediation models did yield several interesting findings (Additional File 4, Table 6). For instance, the total effect of TSC condition on PROMIS-SD at post-treatment, as well as PROMIS-SRI at post-treatment and 6 FU were significant in some of the mediation models, such that Adapted TSC, relative to Standard, was associated with improved sleep and circadian functioning. Also, higher provider ratings of fit were associated with better patient outcomes, regardless of the TSC condition. Although these are not prespecified analyses, such findings suggest that there may be differences worth exploring in future research.

For the exploratory aims, Adapted TSC was associated with a significant decrease in the number of cigarettes per day from pre-treatment to six-month follow-up (Pre: *M* = 11.18, *SD* = 8.3; 6 FU: *M* = 6.78, *SD* = 6.6), relative to Standard TSCC (Pre: *M* = 8.83, *SD* = 7.38; 6 FU: *M* = 9.23, *SD* = 8.95). While the mean number of cigarettes per day for Adapted TSC was higher at the pre-treatment assessment, relative to the Standard TSC, the reduction by 4.4 cigarettes per day in Adapted is not trivial and adds another angle to existing evidence showing associations between smoking cigarettes and poor sleep (e.g., [84]). Finally, there were no differences between Adapted versus Standard TSC on patient ratings of the credibility of the treatment or patient’s perceived improvement.

There were two domains in which Adapted TSC differed from Standard TSC. First, there were a larger number of providers and patients in Adapted TSC (Providers = 63; Patients = 124) compared to Standard TSC (Providers = 30; Patients = 74). Second, there were significantly more drop-outs from Standard TSC relative to Adapted TSC during the treatment phase (Standard 50% vs. Adapted 25.81%). There are several possible explanations for these findings: (a) perhaps there were differing levels of engagement in counties allocated to Standard compared to Adapted and/or (b) perhaps Adapted TSC was more appealing to counties, providers and patients.

As noted earlier, facilitation is the active ingredient within the i-PARIHS framework. Thus, the present study adds to the evidence that external facilitation, by university-based facilitators, is an effective approach to implementation that promotes the implementation of complex EBPTs, like TSC, into complex routine practice (e.g., [28, 50, 51]). In the study described here—and consistent with the i-PARIHS framework [8] and guidelines [52, 53]—facilitation unfolded flexibly and differently depending on the unique challenges and obstacles faced by each provider and each site. There were many opportunities for facilitation and the type and intensity of facilitation varied as a function of differences between sites in their culture, values, resources, extent of leadership support and engagement as well as the extent of provider capacity and willingness. Facilitation was largely focused on supporting the providers to deliver TSC, with some facilitation also focused on the meso and macro contexts. For example, the process of facilitation uncovered that offering CE credits for TSC training was highly motivating for providers and facilitation was needed to work out the details how TSC sessions could be counted as billable hours and reimbursed.

Several notes and limitations warrant consideration. First, we did not have the resources or capacity to collect data on specific aspects of the context that are important within i-PARIHS and within the field of implementation science more broadly, such as the outer context nor the dynamic relationships between the micro, meso and macro layers of context [8]. This is an important area for future research, given their established importance in the implementation and sustainment process [85]. Second, as specified in the protocol paper [35], the outcomes from this multi-site, multi-year study will be reported across multiple publications. This study has addressed the pre-specified outcomes for the main aims of the Implementation Phase. Future publications will focus on the Train-the-Trainer and Sustainment Phases. Other important outcomes—such as facilitation tracking—were not pre-specified and warrant separate in-depth publications. Third, there are several aspects that might seem like limitations but are, in fact, essential elements of this study. Specifically, although we trained providers to deliver 4 sessions in Adapted TSC and 8 sessions in Standard TSC, on the ground the Adapted providers delivered a mean of 4.99 sessions (SD = 1.91) and Standard providers delivered a mean of 8.95 sessions (SD = 8.01). This raises the possibility that providers may have believed patients needed more sessions than were originally prescribed. Also, due to the imperative to alleviate patient burden in this large multi-phase and multi-site study, patient self-reported psychiatric diagnoses were collected instead of administering a structured clinical interview. Similarly, it was not realistic to collect sleep diary and actigraphy. While sleep diary and actigraphy are gold standard measures for sleep research, they are not considered “essential” for “treatment studies focusing on large community samples in routine practice settings” (p. 1156; [86]). Fourth, the design of the study precluded a comparison between conditions for the main outcomes at the 6-month follow-up. Also, we acknowledge that the UC-DT design may inflate the effect size differences [87] and that corrections for multiple secondary outcomes were not conducted. The secondary outcomes are not considered confirmatory, replication is needed. Fifth, while the present study monitored adverse events, adverse events should be pre-specified, pre-defined and measured [88]. This is particularly vital in sleep treatments as adverse events have been documented [88]. Sixth, the SDS is scored by summing the responses. However, participants who did not work or attend school skipped item 1: “The symptoms have disrupted your work/school.” Because higher SDS scores reflect greater impairment, the omission of this item may have artificially lowered the SDS scores. Also, the measure of fit we employed involved the providers rating items on a 1 (completely disagree) to 5 (completely agree) scale. As evident in Table 1, the mean ratings were above 4.5. Thus, a ceiling effect may have precluded group differences from emerging. Future research to refine the measurement of fit in a non-burdensome way is recommended. Notably, if fit was indicated by administrative metrics, like recruitment success or provider participation rates (which may imply feasibility and acceptability of the treatment and potential uptake), Adapted TSC shows better fit than Standard TSC. Seventh, it is possible that baseline differences in government assistance and education may have influenced the results, although these differences only trended toward significance. Finally, it was not possible to distinguish drop-outs due to a patient discontinuing treatment from other causes, such as providers not having time to deliver an adequate number of treatment sessions. Clearly differentiating causes of drop-outs will be essential for future research.

## Conclusions

Both the Adapted and Standard versions of TSC were associated with improvement in sleep, psychiatric symptoms and functional impairment. These findings add to the growing support for the Sleep Health Framework (e.g., [10, 89, 90]), REP (e.g., [91–94]), the i-PARIHS framework [8] and the use of external facilitation as an implementation strategy (e.g., [28, 50, 51]). This study was conducted in a community mental health setting and TSC was delivered by busy CMHC providers. As such, the findings constitute a meaningful step toward bridging the large gap between research and routine practice. Importantly, the use of theory, data and end-user perspectives to guide the adaptation of TSC responds to the surge of interest in increasing the scientific rigor of the treatment adaptation process [33, 38, 95] and yielded a range of interesting insights including that both Adapted and Standard TSCC are valuable approaches. Overall, the fact that Adapted TSC is as effective and similarly rated as acceptable, feasible, and appropriate compared to Standard TSC is promising for supporting the use of a briefer version in under-resourced settings. These findings can be interpreted through the lens of the four core constructs of the i-PARIHS framework: adapting an innovation to contextual needs and delivering it through facilitation can be acceptable and effective for provider and patient recipients. The extent to which these findings are specific to CMHCs or generalizable to other contexts should be evaluated in future research.

## Supplementary Information

Additional File 1

## Data Availability

Raw data for most outcomes reported herein has been uploaded into the NIMH National Data Archive.

## Abbreviations

6FU: Six months after treatment
AIM: Acceptability of Intervention Measure
ANCOVA: Analysis of covariance
CBT: Cognitive behavioral therapy
CEQ: Credibility/Expectancy Questionnaire
CMHCs: Community mental health centers
DSM-5: DSM-5 Cross-Cutting Measure
EBPT: Evidence-based psychological treatments
FIM: Feasibility of Intervention Measure
i-PARIHS: Integrated Promoting Action on Research Implementation in Health Services framework
IAM: Intervention Appropriateness measure
ICC: Intraclass correlation coefficients
MDES: Minimum detectable effect sizes
MLMs: Multilevel models
MP: Mediated proportions
PROMIS-SD: PROMIS-Sleep Disturbance
PROMIS-SRI: PROMIS-Sleep Related Impairment
REP: Replicating Effective Programs
SDS: Sheehan Disability Scale
SE: Robust standard errors
SEMs: Structural equation models
SHC: Sleep Health Composite
SMI: Serious mental illness
SNAP: Supplemental Nutrition Assistance Program
SSI/SSDI: Supplemental Social Security Income/Social Security Disability Insurance
UC-DT: Usual Care followed by Delayed Treatment with TSC

## Footnotes

1 Previously referred to as TranS-C.

2 Note that the vast majority of providers and patients were employed by or seeking/receiving treatment at CMHCs. However, in very few isolated instances, providers and patients outside of CMHCs learned about the study (e.g., by word of mouth) and requested to participate. When the providers or patients otherwise met the criteria, they were permitted to participate, and were matched with a CMHC patient or provider, respectively, by the facilitation team.

3 This approach was taken when the patient and provider were uncertain about the SMI diagnosis.

4 Due to administrative error, the 16-item version was originally administered. However, standardized T-scores are only available for the 8-item version. Thus, only the 8 items that comprise the 8-item version were included in the scoring and analyses.

5 This approach to mediation deviates from the approach described in the protocol paper (i.e., multilevel SEMs). This change was made, because there is limited published guidance on using multilevel SEM for mediation in longitudinal RCTs. Although our syntax for multilevel SEMs produced the same pattern of results as those produced with the ANCOVA approach, we felt more confident in the estimates yielded for the latter.
