# Supplementary material for: A randomized trial of adapted versus standard versions of the Transdiagnostic Intervention for Sleep and Circadian Dysfunction implemented via facilitation and delivered by community mental health providers: improving the “fit” of psychological treatments by adapting to context
Source: Implement Sci. 2025 Jul 9;20:32. doi: 10.1186/s13012-025-01440-9 (PMC12239326; doi:10.1186/s13012-025-01440-9)
Supplement: Supplementary file 1 — Additional File 1 [file 13012_2025_1440_MOESM1_ESM.docx]

**Additional File 1: Further information on treatments delivered**

**Facilitation**

As described in the protocol paper (1), the specific activities of the facilitators included the following. First, the facilitators organized and lead regular TSC trainings for interested CMHC providers. Second, the facilitators distributed treatment manuals and workbooks to all participating providers and patients. Third, drop-in supervision was offered once a week separately for each condition by the lead facilitator (ERA) and a clinical science graduate student (CAC) with expertise in TSC and SMI. Fourth, consultation was provided on an ongoing, as-needed basis by the facilitators via phone calls, text messaging, and emails. Fifth, the facilitators organized and lead regular presentations to the CMHCs on advanced topics related to sleep and mental health (e.g., Lunch & Learn, Coffee Colloquium, Booster Sessions). Sixth, facilitators helped with administrative barriers, such as working to ensure that TSC trainings count toward Continuing Education credits. Seventh, facilitators offered a sleep treatment certification that CMHC providers can achieve via three supervised TSC cases. Eighth, facilitators maintained an active website with supplemental treatment-related resources for CMHC providers. Ninth, facilitators developed and distributed materials (e.g., flyers, educational videos, social media posts) related to sleep knowledge and mental health at the request of CMHC leadership and providers. Tenth, facilitators held regular and as-needed meetings with CMHC leadership and key providers to provide progress updates, collaborate on decision-making, and problem-solve barriers to effective intervention delivery such as organizational burnout, site cultures that are resistant to change, lack of resources or funding, oversaturation of treatment options, and logistical barriers. Eleventh, facilitators identified and cultivated intervention “champions” who could model effective treatment delivery and support their colleagues in using TSC.

**Transdiagnostic Intervention for Sleep and Circadian Dysfunction (TSC)**

TSC is considered to be “transdiagnostic” in two ways (2): it targets a variety of sleep and circadian diagnoses commonly experienced by individuals diagnosed with a range of SMIs, and it offers a single, brief protocol to address sleep and circadian problems that don't fit neatly into specific categories. TSC was designed to promote sleep health along the six dimensions specified in the Sleep Health Framework (3).

**Standard TSC**

“Standard” TSC is a modular approach, comprised of (a) four core modules that form the basic building blocks of sleep health, (b) four cross-cutting interventions used in every session (e.g., motivational enhancement), and (c) seven optional modules that can be integrated based on case conceptualization, patient goals, and clinical judgment. “Standard” TSC involves relatively high-intensity procedures like other EBPTs, including eight weekly, 50-minute sessions

The *cross-cutting modules* were case formulation, sleep and circadian education, motivational enhancement, and goal setting. *Core module 1* targets irregular sleep-wake times, difficulty winding-down, and difficulty waking-up. *Core module 2* aims to reduce daytime impairment*.* *Core module 3* focuses on unhelpful beliefs about sleep*. Core module 4* aims to promote maintenance of changes made during treatment. *Optional module 1* addresses poor sleep efficiency via stimulus control (4) and sleep restriction (5). *Optional module 2* helps patients reduce time in bed. *Optional module 3* addresses delayed or advanced phase problems (e.g., going to sleep later than desired or waking up earlier than desired). *Optional module 4* helps patients manage worries about sleep. *Optional Module 5* promotes compliance with Continuous Positive Airways Pressure (CPAP) for patients with sleep apnea. *Optional Module 6* helps patients negotiate sleep in complicated environments (e.g., noise from bed/roommates, traffic noise, streetlight entering the bedroom). *Optional Module 7* was for patients who experience nightmares.

**Adapted TSC**

As described in the protocol paper (1), there have been calls for rigorous approaches to treatment adaptation (6-8). In response, we grounded the process for adapting TSC in theory, data, and end-user input. As the overarching guide for the adaptation process, the Replicating Effective Programs (REP) framework (9) was used. Phase 1 of REP (Pre-Condition) was completed prior to the present protocol. First, as discussed above, we established that there is a need for effective, feasible EBPTs for SMI in CMHCs and that sleep and circadian functioning may represent a powerful target to help address this need. Second, we determined that there was empirical support for TSC in CMHCs (10). Third, we gathered end-user input on fit and packaging of the intervention (11, 12). Fourth, we reviewed past data and identified the TSC treatment skills that were most utilized by patients with a utilization scale adapted from Gumport et al. (13). Fifth, we considered TSC’s theoretical underpinnings and mechanisms of action (2, 3) from which we retained the core elements (6, 7, 14). Sixth, we piloted Adapted TSC with 21 adults through the PI’s UC Berkeley research clinic (unpublished data). Informal feedback was solicited from providers and patients who participated in this pilot to further refine Adapted TSC. In Phase 2 of REP (Pre-Implementation), we customized the delivery of TSC training and treatment materials to the CMHC context based on the input from CMHC leadership, staff, and patients (Armstrong et al., 2022; Gumport et al., 2020). Throughout REP Phases 1 and 2, following leading adaptation frameworks, we sought to ensure that Adapted TSC would be relevant to the broadest range of patients and to account for factors that impact implementation (e.g., resources required) (7, 15, 16). The present trial addressed the last two phases of REP – namely, Phases 3 (Implementation) and 4 (Maintenance and Evolution).

Adapted TSC consisted of the same four *cross-cutting and core modules* as in Standard TSC, but the core modules were split up into four modules. *Core module 1* targeted irregular sleep-wake times. *Core module 2* targeted difficulty winding down. *Core module 3* targeted difficulty waking up. *Core module 4* aimed to reduce daytime impairment. *Core module 5* promoted maintenance of change. The one *optional module* focused on reducing sleep-related worry and can be integrated with the core modules, based on clinical presentation, treatment goals, and provider case conceptualization.

Note that Adapted TSC consists of the same core modules as Standard TSC, except that Unhelpful Beliefs about Sleep is not included. This is a clarification of our protocol papers for this study (Sarfan et al., 2023; Callaway et al., 2023) in which we stated that the core modules were the same as Standard TSC, but split up into five, rather than four modules. Cosmetically, as the project evolved, we relabeled the core module numbers in the Adapted TSC condition as “1, 2 and 3” versus the Standard TSC labels of “1a, 1b, and 1c” to help the providers follow that these were separate modules to be delivered in different sessions.

**Additional File 2: Further information on measures and methods**

**PhenX Toolkit: Substance Use and Suicidality**.

Scales from the PhenX Toolkit (17) were used to assess various patient outcomes. To assess suicidal ideation and behaviors, the PhenX ‘Classification of Suicidal Ideation and Suicidal Behavior - Adult - Current’ protocol was used. This protocol includes two subscales from the screening version of the Columbia-Suicide Severity Rating Scale: Severity of Suicidal Ideation and Suicidal Behavior, assessing suicidality during two time periods—namely ideation in the past month and suicidal behavior in the past three months. To ease patient burden, this measure was adapted slightly, such that if patients denied suicidal ideation, they were not required to answer questions about suicidal behavior. These scales were scored according to the scoring guide (18). Specifically, the suicidal ideation scale was scored such that each question was assigned a sequential, numerical value ranging from 1 to 5 as the questions increased in severity (Question 1: “Have you wished you were dead or wished you could go to sleep and not wake up?” = 1, to Question 5: "Have you started to work out or worked out the details of how to kill yourself? Do you intend to carry out this plan?” = 5). The highest numerical value (i.e., the value associated with the most severe item endorsed) was used as the final score. Participants were given a 0 if no ideation was endorsed. For suicidal behavior, participants were asked whether they had engaged in five suicide-related behaviors in the past three months (i.e., actual attempt, aborted attempt, interrupted attempt, preparatory behavior/s, and/or nonsuicidal self-injury). Each item was reported on a binary scale (0 = no, 1 = yes) and frequency of patients who endorsed a given behavior was identified.

To assess alcohol, select questions from the PhenX ‘Alcohol - 30-Day Quantity and Frequency’ protocol were administered. Following prior research (e.g., 19), number of days on which patients drank alcohol in the past 30 days was used as the outcome.

To assess tobacco, the PhenX ‘Tobacco - 30-Day Quantity and Frequency - Adult' protocol was used. This measure has three sets of question protocols: (1) a protocol for ‘Every-Day Smokers,’ (2) a protocol for ‘Some-Day Smokers,’ and (3) a protocol for ‘Former Smokers.’ If patients reported that they had never smoked tobacco, this measure was skipped. Following prior research, the outcome computed for the present study was average cigarettes smoked per day (CPD) (e.g., 20). Specifically, if patients reported that they smoked ‘every day’ at the time of assessment, they were asked to report the average number of cigarettes that they smoked per day. If patients reported that they smoked on ‘some days’ at the time of assessment, they were asked to report the number of days they smoked cigarettes in the past 30 days and, on average on those days, how many cigarettes they smoked. An average CPD was computed collapsing across these two groups of patients.

Caffeine was assessed using questions adapted from the ‘Supplemental Beverage Questionnaire.’ The original measure suggested calculating the caffeine milligrams ingested per year (21). However, to reduce participant burden and better capture the timeframes assessed in the present study, some questions from the original measure were not administered – in particular, the type of caffeinated drink consumed (used to calculate annual caffeine servings in the original measure). Thus, number of caffeinated drinks per day, on average, over the past 30 days was used as the outcome.

To assess use of other psychoactive substances, the PhenX ‘Substances - 30-Day Frequency’ protocol was used. This measure assesses the number of days on which participants used substances such as sedatives, painkillers, stimulants, and hallucinogens over the past 30 days. Following past research (22-24), a binary variable was created by collapsing across substances to indicate use of any of these substances over the past 30 days (0 = no use of a psychoactive substance over the past 30 days, 1 = use of a psychoactive substance over the past 30 days).

**Recruitment**

Building the CMHC network that formed the basis for this study began in August 2013 with outreach by the PI. The network has been maintained via newsletters, meetings, and workshops on EBPTs. Originally, eight counties agreed to participate. At various stages of the study, we recruited new counties and new CMHC sites to maximize provider and patient sample size goals. Most counties consisted of three to 10 CMHC sites. Sites in the following ten counties in California, United States participated in the Implementation Phase: Alameda, Contra Costa, Kings, Monterey, Placer, Santa Cruz, Solano, Santa Clara^[[1]](#footnote-1)^, Santa Barbara, and Lake. Note that sites in San Luis Obispo also participated but are operating as part of Monterey County.

To recruit providers, UC Berkeley facilitators met with key CMHC leadership, who helped to engage and recruit providers in their CMHC. Providers were also recruited through flyers posted in CMHCs, announcements at staff meetings, meetings organized by the facilitators, and appointments by leadership. During the TSC trainings, facilitators continued to engage and recruit providers by describing the benefits of participating in the study. After TSC trainings, facilitators followed up with weekly emails for one month to encourage providers to participate.

Patients were recruited through a variety of methods, based on each CMHC’s preference. These methods include the following: (1) posting flyers from the research team in waiting rooms and providers’ offices; (2) integrating a sleep screener into intake paperwork; (3) asking providers to screen patients on their caseload; and (4) encouraging word of mouth between patients. Potentially eligible patients were typically identified by their provider. After eligibility was confirmed and consent to participate in the study was given, the patient was matched to a CMHC TSC provider.

**Additional File 3: Further information on data analysis**

**Assumptions Checks**

Model assumptions for multilevel models (MLMs), structural equation models (SEMs), and linear regressions models were evaluated. For MLMs and linear regressions, qqplots and histograms of model residuals were examined. Additionally, Levene’s test was used to formally test for homogeneity. For SEMs, most models were saturated, resulting in a null residual matrix. Thus, the normality and homogeneity of the residuals for each path were evaluated. In some models, violations of normality and homoscedasticity were detected. Therefore, across all models, robust standard errors (‘sandwich estimators’) were used, which are robust to violations of assumptions, particularly homoscedasticity (25).

***Missing Data***

Per the protocol paper, we tested whether missingness was related to other, theoretically-plausible predictors, which would suggest that the data were missing at random (25-27).First, we evaluated whether the following observed variables predicted missingness of *patient* data at post-treatment: treatment condition (UC-DT vs. immediate and Adapted vs. Standard; 28), patient sex (given its relationship to sleep problems; e.g., 29), other patient sociodemographics ((e.g., income, education, race, ethnicity, government assistance, employment status; 30), patient variables used for stratification (age, substance use, psychosis; 31), and provider theoretical orientation (CBT or other; e.g., 32, 33), prior training in sleep problems (32), and perceptions of TSC fit at pre, mid, and post (given that treatment fit can predict providers’ ongoing use of a given treatment; e.g., 34). Second, we tested whether the following observed variables predicted missingness of *provider* data at post-treatment: treatment condition (Adapted vs. Standard; Rabe-Hesketh & Skrondal, 2012), variables that have been found to predict provider perceptions or use of treatment (i.e., CBT orientation, prior training in sleep problems; Addis & Krasnow, 2000; Garcia et al., 2020), and patient symptom severity at pre-treatment (based on qualitative data that this impacted provider use or discontinuation of TSC) (35). Third, we tested whether the planned covariate of county predicted missingness for patients and providers (1).

To test these possibilities, we created a dummy-coded variable for all patients to indicate whether they had missing data or no missing data at post-treatment (1 = missing data; 0 = no missing data). We then used logistic regression or chi-square tests, depending on whether the predictor was continuous or categorical, respectively, to test whether any of these observed variables predicted missingness group membership (i.e., whether patients had missing data or did not have missing data).

Findings from these analyses suggested that patients’ treatment condition assignments were associated with missing data at post-treatment, such that patients in UC-DT (vs. immediate TSC) and Adapted (vs. Standard) TSC had less missing data. Patients who were female (vs. male) had less missing data. Additionally, patients who did not have children (vs. those who did) had less missing data. Of the provider variables, prior training in sleep problems (vs. no prior training) and higher perceptions of feasibility and appropriateness at mid-treatment significantly predicted less missing patient data at post-treatment. For providers, only treatment condition predicted missing data, such that Adapted (vs. Standard) was associated with less missing data. These results for providers and patients lend support to the data being missing at random, in that missing data appeared to be associated with theoretically-grounded observed variables (Black et al., 2017; Grund et al., 2019; Rabe-Hesketh & Skrondal, 2012). As specified in the protocol paper, these variables were included in the relevant models as covariates, except when they were already tested as predictors in a given model.

**Covariates**

Per the protocol paper, covariates tested included planned variables and variables identified during the aforementioned missing data analyses (1). For Aim 1, the covariates included patient TSC condition *(identified from missing data analyses)*, patient stratification variables (age, substance use, psychosis; *planned*), patient sex *(from missing data analyses)*, patient number of children *(from missing data analyses*) and provider prior training in sleep problems, FIM at mid and IAM at mid *(all from missing data analyses)*. For Aim 2, the covariate tested was county *(planned)*. For Aim 3 and Exploratory Aims 1 and 2, the covariates were the same as Aim 1, except when variables were already included as a predictor or mediator.

For patient models, collinearity prevented county and TSC condition to be run simultaneously. Thus, two sets of covariate models were run: one with county and one with TSC condition. The pattern of results were the same, with the exception of an indirect effect for the Aim 1 SEM analyses, which is noted in the main paper results. In the final patient models, we selected the models with TSC condition (vs. county) along with the other covariates, because it had predicted missing data, whereas county had not. County was retained as a covariate for provider analyses. Note that, running models with (vs. without) covariates did not change the pattern of results for the primary parameters of interest (i.e., time-by-treatment interactions in MLMs, indirect effects in SEMs), except for the Aim 1 SEM mentioned above.

**References cited**

1. Sarfan LD, Agnew ER, Diaz M, Dong L, Fisher K, Spencer JM, et al. The Transdiagnostic Intervention for Sleep and Circadian Dysfunction (TranS-C) for serious mental illness in community mental health part 1: study protocol for a hybrid type 2 effectiveness-implementation cluster-randomized trial. Trials. 2023;24(1):1-18.

2. Harvey AG, Buysse DJ. Treating Sleep Problems: A Transdiagnostic Approach: Guilford Publications; 2017.

3. Buysse DJ. Sleep health: can we define it? Does it matter? Sleep. 2014;37(1):9-17.

4. Bootzin RR. Stimulus control treatment for insomnia. Proceedings of the American Psychological Association. 1972;7:395-6.

5. Spielman AJ, Caruso LS, Glovinsky PB. A behavioral perspective on insomnia treatment. Psychiatric Clinics of North America. 1987;10:541-53.

6. Escoffery C, Lebow-Skelley E, Haardoerfer R, Boing E, Udelson H, Wood R, et al. Systematic review of adaptations of public health evidence-based interventions. Implementation Science. 2018;13(1):125.

7. Escoffery C, Lebow-Skelley E, Udelson H, Böing EA, Wood R, Fernandez ME, et al. A scoping study of frameworks for adapting public health evidence-based interventions. Translational Behavioral Medicine. 2018;9(1):1-10.

8. Harvey AG, Lammers H, Dolsen MR, Tran M, Tuck A, Hilmoe H, et al. Advancing the science of treatment adaptation and ad hoc adaptations of cognitive, behavioral and related evidence-based psychosocial treatments for adults diagnosed with a mental illness: a systematic review. . Evidence-Based Meantl Health. 2020.

9. Kilbourne AM, Neumann MS, Pincus HA, Bauer MS, Stall R. Implementing evidence-based interventions in health care: application of the replicating effective programs framework. Implementation Science. 2007;2(1):1-10.

10. Harvey AG, Dong L, Hein K, Yu S, Martinez A, Gumport N, et al. A randomized controlled trial of the Transdiagnostic Intervention for Sleep and Circadian Dysfunction (TranS-C) to improve serious mental illness outcomes in a community setting. Journal of Consulting and Clinical Psychology. 2021;89(6):537-44.

11. Armstrong CC, Harvey AG. Barriers and facilitators to behavior change for individuals with severe mental illness who received the transdiagnostic intervention for sleep and circadian dysfunction in a community mental health setting. The Journal of Behavioral Health Services & Research. 2021:1017.

12. Gumport NB, Yu SH, Harvey AG. Implementing a transdiagnostic sleep and circadian intervention in a community mental health setting: A qualitative process evaluation with community stakeholders. Psychiatry Research. 2020;293:113443.

13. Gumport NB, Dolsen MR, Harvey AG. Usefulness and Utilization of Treatment Elements from the Transdiagnostic Sleep and Circadian Intervention with Adolescents with an Evening Circadian Preference. Behavior Research and Therapy. 2019;123:103504.

14. Lee SJ, Altschul I, Mowbray CT. Using planned adaptation to implement evidence‐based programs with new populations. American Journal of Community Psychology. 2008;41(3-4):290-303.

15. Aarons GA, Green AE, Palinkas LA, Self-Brown S, Whitaker DJ, Lutzker JR, et al. Dynamic adaptation process to implement an evidence-based child maltreatment intervention. Implementation Science. 2012;7(1):32.

16. Weisz JR. Building robust psychotherapies for children and adolescents. Perspectives on Psychological Science. 2014;9(1):81-4.

17. Hamilton CM, Strader LC, Pratt JG, Maiese D, Hendershot T, Kwok RK, et al. The PhenX Toolkit: get the most from your measures. American journal of epidemiology. 2011;174(3):253-60.

18. Nilsson ME, Suryawanshi S, Gassmann-Mayer C, Dubrava S, McSorley P, Jiang K. Columbia-Suicide Severity Rating Scale Scoring and Analysis Guide (Version 2.0). Retrieved from <https://cssrs.columbia.edu/wp-content/uploads/ScoringandDataAnalysisGuide-for-Clinical-Trials-1.pdf2013>.

19. Hinojosa CA, Liew A, An X, Stevens JS, Basu A, Van Rooij SJ, et al. Associations of alcohol and cannabis use with change in posttraumatic stress disorder and depression symptoms over time in recently trauma-exposed individuals. Psychological medicine. 2024;54(2):338-49.

20. Sakuma K-LK, Pierce JP, Fagan P, Nguyen-Grozavu FT, Leas EC, Messer K, et al. Racial/ethnic disparities across indicators of cigarette smoking in the era of increased tobacco control, 1992–2019. Nicotine and Tobacco Research. 2021;23(6):909-19.

21. Song YJ, Kristal AR, Wicklund KG, Cushing-Haugen KL, Rossing MA. Coffee, tea, colas, and risk of epithelial ovarian cancer. Cancer Epidemiology Biomarkers & Prevention. 2008;17(3):712-6.

22. Brown JL, Cochran G, Bryan MA, Charron E, Winhusen TJ. Associations between elevated depressive symptoms and substance use, prescription opioid misuse, overdose history, pain, and general health among community pharmacy patients prescribed opioids. Substance abuse. 2022;43(1):1110-5.

23. Gerke DR, Call J, Auslander WF. The syndemic factors of violence exposure, Substance Use, and Mental Health problems: Relationships to sexual risk behaviors in HIV-Negative Young Men who have sex with men. Journal of the Society for Social Work and Research. 2022;13(2):235-59.

24. Shmulewitz D, Stohl M, Greenstein E, Roncone S, Walsh C, Aharonovich E, et al. Validity of the DSM-5 craving criterion for alcohol, tobacco, cannabis, cocaine, heroin, and non-prescription use of prescription painkillers (opioids). Psychological medicine. 2023;53(5):1955-69.

25. Rabe-Hesketh S, Skrondal A. Multilevel and longitudinal modeling using Stata (3rd ed.): Stata Press; 2011.

26. Black AC, Harel O, Betsy McCoach D. Missing data techniques for multilevel data: Implications of model misspecification. Journal of Applied Statistics. 2011;38(9):1845-65.

27. Grund S, Lüdtke O, Robitzsch A. Missing data in multilevel research. In: Humphrey SE, LeBreton JM, editors. The handbook of multilevel theory, measurement, and analysis (pp 365-396): American Psychological Association; 2017.

28. Rabe-Hesketh S, Skrondal A. Multilevel and Longitudinal Modeling using Stata-Volume II: Categorical Responses. Counts, and Survival, StataCorp LP, College Station, TX. 2012.

29. Zhang B, Wing Y-K. Sex differences in insomnia: a meta-analysis. Sleep. 2006;29(1):85-93.

30. Hale L, Troxel W, Buysse DJ. Sleep health: An opportunity for public health to address health equity. Annual review of public health. 2020;41:81-99.

31. Kahan BC, Morris TP. Reporting and analysis of trials using stratified randomisation in leading medical journals: review and reanalysis. British Medical Journal. 2012;345.

32. Garcia HA, Mignogna J, DeBeer BR, Song J, Haro EK, Finley EP. Provider factors predict use of evidence-based psychotherapies in veterans affairs posttraumatic stress disorder specialty programs: The role of profession, theoretical orientation, and training. Traumatology. 2020;26(2):227.

33. Addis ME, Krasnow AD. A national survey of practicing psychologists' attitudes toward psychotherapy treatment manuals. Journal of consulting and clinical psychology. 2000;68(2):331.

34. Barnett M, Brookman-Frazee L, Yu SH, Lind T, Lui J, Timmer S, et al. Train-to-sustain: Predictors of sustainment in a large-scale implementation of parent–child interaction therapy. Evidence-based practice in child and adolescent mental health. 2021;6(2):262-76.

35. Sarfan LD, Bajwa Z, Diaz M, Tiab S, Fisher K, Agnew ER, et al. “So Many Other Things Improve” with Transdiagnostic Treatment for Sleep and Circadian Problems: Interviews with Community Providers on Treating Clients with Serious Mental Illness. Administration and Policy in Mental Health and Mental Health Services Research. 2024:1-13.

**Additional File 4: Further results**

**Supplement Table 1. Percent of Missing Data for Aim 1 Measures by Treatment Condition and Timepoint**

|  | UC-DT (*n* = 198) | | | | | | TSC (*n* = 198) | | | | Total (*N* = 396) | | | | |  |  |
| --- | --- | --- | --- | --- | --- | --- | --- | --- | --- | --- | --- | --- | --- | --- | --- | --- | --- |
|  | Pre | | Post | | | Pre | | | Post | | Pre | | Post | | | |  |
|  | n | % | n | % | n | | | % | n | % | n | % | | n | % | | |
| **Outcome** |  |  |  |  |  | | |  |  |  |  |  | |  |  | | |
| PROMIS-SD | 0 | 0 | 21 | 10.61 | 1 | | | 0.51 | 95 | 47.98 | 1 | 0.25% | | 116 | 29.29% | | |
| PROMIS-SRI | 0 | 0 | 21 | 10.61 | 3 | | | 1.52 | 96 | 48.48 | 3 | 0.76% | | 117 | 29.55% | | |
| SHC | 16 | 8.08 | 42 | 21.21 | 19 | | | 9.60 | 100 | 50.51 | 35 | 8.84% | | 142 | 35.86% | | |
| SDS | 0 | 0 | 21 | 10.61 | 1 | | | 0.51 | 95 | 47.98 | 1 | 0.25% | | 116 | 29.29% | | |
| DSM-5 | 0 | 0 | 22 | 11.11 | 6 | | | 3.03 | 96 | 48.48 | 6 | 1.52% | | 118 | 29.80% | | |
| *Note.* PROMIS-SD = PROMIS Sleep Disruption. PROMIS-SD = PROMIS Sleep Disturbance. PROMIS-SRI = PROMIS Sleep-Related Impairment. SHC = Sleep Health Composite. DSM-5 = DSM-5 Cross-Cutting. SDS = Sheehan Disability Scale. TSC = Transdiagnostic Intervention for Sleep and Circadian Dysfunction. UC-DT = usual care followed by delayed treatment with TSC. | | | | | | | | | | | | | | | |  |  |

**Supplement Table 2. Percent of Missing Data for Aim 2 Measures by Treatment Condition and Timepoint**

|  | Adapted | | | | Standard | | | | Total | | | | |
| --- | --- | --- | --- | --- | --- | --- | --- | --- | --- | --- | --- | --- | --- |
|  | Pre  (*n* = 63) | | Post  (*n* = 124) | | Pre  (*n* = 30) | | Post  (*n* = 74) | | Pre  (*n* = 93) | | Post  (*n* = 198) | |  |
|  | N | % | n | % | n | % | n | % | n | % | n | % |  |
| **Outcome** |  |  |  |  |  |  |  |  |  |  |  |  |  |
| AIM | 12 | 19.05 | 25 | 20.16 | 2 | 6.67 | 32 | 43.24 | 14 | 15.05 | 57 | 28.79 |  |
| IAM | 12 | 19.05 | 25 | 20.16 | 2 | 6.67 | 32 | 43.24 | 14 | 15.05 | 57 | 28.79 |  |
| FIM | 12 | 19.05 | 25 | 20.16 | 2 | 6.67 | 32 | 43.24 | 14 | 15.05 | 57 | 28.79 |  |
| *Note.* AIM = Acceptability of Intervention Measure. FIM = Feasibility of Intervention Measure. IAM = Intervention Appropriateness measure. ‘Standard’ and ‘Adapted’ indicate TSC condition. Each provider only completed the pre-treatment AIM, IAM, FIM one time. They were asked to complete these measures at post for each patient (which is why the post-treatment sample sizes are larger than pre-treatment). Providers were not asked to complete these measures at 6FU. | | | | | | | | | | | | | |

**Supplement Table 3. Percent of Missing Data for Aim 3 Measures by Treatment Condition and Timepoint**

|  | Adapted | | | | | | Standard | | | | | | | | Total | | | | | | | | |
| --- | --- | --- | --- | --- | --- | --- | --- | --- | --- | --- | --- | --- | --- | --- | --- | --- | --- | --- | --- | --- | --- | --- | --- |
| **Provider Outcomes** | Pre  (n = 63) | | Post  (n = 124) | | 6FU  (n = 124) | | Pre  (n = 30) | | Post  (n = 74) | | | 6FU (n = 74) | | | Pre  (n = 93) | | | Post  (n = 198) | | | 6FU  (n = 198) | | |
|  | *n* | % | *n* | % | *n* | % | *n* | % | *n* | % | *n* | | % | *n* | | % | *n* | | % | *n* | | % | |
| AIM | 12 | 19.05 | 25 | 20.16 | n/a | n/a | 2 | 6.67 | 32 | 43.24 | n/a | | n/a | 14 | | 15.05 | 57 | | 28.79 | n/a | | n/a | |
| IAM | 12 | 19.05 | 25 | 20.16 | n/a | n/a | 2 | 6.67 | 32 | 43.24 | n/a | | n/a | 14 | | 15.05 | 57 | | 28.79 | n/a | | n/a | |
| FIM | 12 | 19.05 | 25 | 20.16 | n/a | n/a | 2 | 6.67 | 32 | 43.24 | n/a | | n/a | 14 | | 15.05 | 57 | | 28.79 | n/a | | n/a | |
| **Patient Outcomes** | Pre  (n = 124) | | Post  (n = 124) | | 6FU  (n = 124) | | Pre  (n = 74) | | Post  (n = 74) | | | 6FU  (n = 74) | | | Pre  (n = 198) | | | Post  (n = 198) | | | 6FU  (n = 198) | | |
|  | *n* | % | *n* | % | n | % | *n* | % | *n* | % | *n* | | % | *n* | | % | *n* | | % | *n* | | % | |
| PROMIS-SD | 0 | 0 | 46 | 37.10 | 23 | 18.55 | 1 | 1.35 | 49 | 66.22 | 28 | | 37.84 | 1 | | 0.51 | 95 | | 47.98 | 51 | | 25.76 | |
| PROMIS-SRI | 2 | 1.61 | 46 | 37.10 | 24 | 19.35 | 1 | 1.35 | 50 | 67.57 | 28 | | 37.84 | 3 | | 1.52 | 96 | | 48.48 | 52 | | 26.26 | |
| SDS | 0 | 0 | 46 | 37.10 | 23 | 18.55 | 1 | 1.35 | 49 | 66.22 | 29 | | 39.19 | 1 | | 0.51 | 95 | | 47.9 | 52 | | 26.26 | |
| DSM-5 | 4 | 3.23 | 46 | 37.10 | 26 | 20.97 | 2 | 2.7 | 50 | 67.57 | 31 | | 41.89 | 6 | | 3.03 | 96 | | 48.4 | 57 | | 28.78 | |
| *Note.* AIM = Acceptability of Intervention Measure. FIM = Feasibility of Intervention Measure. IAM = Intervention Appropriateness measure. PROMIS-SD = PROMIS Sleep Disruption. PROMIS-SD = PROMIS Sleep Disturbance. PROMIS-SRI = PROMIS Sleep-Related Impairment. DSM-5 = DSM-5 Cross-Cutting. SDS = Sheehan Disability Scale. ‘Standard’ and ‘Adapted’ indicate TSC condition. Pre = pre-treatment assessment. Post = post-treatment assessment. 6FU = six-month follow-up assessment. Each provider only completed the pre-treatment AIM, IAM, FIM one time. They were asked to complete these measures at post for each patient (which is why the post-treatment sample sizes are larger than pre-treatment). Providers were not asked to complete these measures at 6FU. | | | | | | | | | | | | | | | | | | | | | | |  |

**Supplement Table 4. Frequency of PhenX-Assessed Suicidal Behaviors and Illicit Substance Use by Timepoint and Treatment Condition**

|  | **Pre-Treatment** | | **Post-Treatment** | | **6FU** | |
| --- | --- | --- | --- | --- | --- | --- |
|  | **Standard** | **Adapted** | **Standard** | **Adapted** | **Standard** | **Adapted** |
| **Outcome** |  |  |  |  |  |  |
| Actual Suicide Attempts | 2 | 1 | 2 | 0 | 1 | 3 |
| Interrupted Suicide Attempts | 0 | 1 | 1 | 0 | 0 | 1 |
| Aborted Suicide Attempts | 1 | 1 | 0 | 1 | 0 | 3 |
| Preparatory Behaviors for Suicide | 3 | 0 | 1 | 2 | 1 | 2 |
| Non-suicidal self-injury | 2 | 1 | 1 | 0 | 1 | 1 |
| Endorsed using illicit substances | 28 | 36 | 7 | 16 | 7 | 24 |
| *Note.* 6FU = six-month follow-up assessment. ‘Standard’ and ‘Adapted’ indicate TSC condition. | | | | | | |

**Supplement Table 5. Pre-Treatment Patient Demographics by Delayed TSC (UC-DT) compared to Immediate TSC**

| **Characteristic** | **UC-DT (*n* = 198)** | | | |  | | **Immediate (*n* = 198)** | | | |  | |  | |  |
| --- | --- | --- | --- | --- | --- | --- | --- | --- | --- | --- | --- | --- | --- | --- | --- |
|  | ***n*** | | ***%*** | |  | | ***n*** | | ***%*** | | **χ^2^** | | ***p*-value** | |  |
| Sex |  | |  | |  | |  | |  | | 0.92 | | 0.63 | |  |
| Female | 117 | | 39.90 | |  | | 125 | | 63.13 | |  | |  | |  |
| Male | 79 | | 59.09 | |  | | 72 | | 36.36 | |  | |  | |  |
| Missing/declined to answer | 2 | | 1.01 | |  | | 1 | | 0.51 | |  | |  | |  |
| Ethnicity |  | |  | |  | |  | |  | | 1.17 | | 0.56 | |  |
| Hispanic or Latino | 75 | | 37.88 | |  | | 66 | | 33.33 | |  | |  | |  |
| Not Hispanic or Latino | 120 | | 60.61 | |  | | 130 | | 65.66 | |  | |  | |  |
| Missing/declined to answer | 3 | | 1.52 | |  | | 2 | | 1.01 | |  | |  | |  |
| Race |  | |  | |  | |  | |  | | 5.34 | | 0.62 | |  |
| American Indian/Alaska Native | 18 | | 9.09 | |  | | 22 | | 11.11 | |  | |  | |  |
| Native Hawaiian/Pacific Islander | 3 | | 1.52 | |  | | 5 | | 2.52 | |  | |  | |  |
| Asian | 22 | | 11.11 | |  | | 14 | | 7.07 | |  | |  | |  |
| Black or African American | 19 | | 9.60 | |  | | 18 | | 9.09 | |  | |  | |  |
| White | 101 | | 51.01 | |  | | 108 | | 54.55 | |  | |  | |  |
| More than one race | 15 | | 7.58 | |  | | 10 | | 5.05 | |  | |  | |  |
| Other/category not listed | 18 | | 9.09 | |  | | 16 | | 8.08 | |  | |  | |  |
| Missing/declined to answer | 2 | | 1.01 | |  | | 5 | | 2.53 | |  | |  | |  |
| Education |  | |  | |  | |  | |  | | 2.75 | | 0.60 | |  |
| High school graduate or below | 57 | | 28.79 | |  | | 61 | | 30.81 | |  | |  | |  |
| Some or completed college or vocational school | 123 | | 62.12 | |  | | 123 | | 62.12 | |  | |  | |  |
| Some or completed graduate school | 17 | | 8.59 | |  | | 11 | | 5.56 | |  | |  | |  |
| Other/category not listed | 0 | | 0.00 | |  | | 1 | | 0.51 | |  | |  | |  |
| Missing/declined to answer | 1 | | 0.51 | |  | | 2 | | 1.01 | |  | |  | |  |
| Employment |  | |  | |  | |  | |  | | 6.10 | | 0.19 | |  |
| Full-time | 32 | | 16.16 | |  | | 27 | | 13.64 | |  | |  | |  |
| Part-time | 27 | | 13.64 | |  | | 38 | | 19.19 | |  | |  | |  |
| Not employed | 126 | | 63.64 | |  | | 124 | | 62.63 | |  | |  | |  |
| Other/category not listed | 13 | | 6.57 | |  | | 7 | | 3.54 | |  | |  | |  |
| Missing/declined to answer | 0 | | 0.00 | |  | | 2 | | 1.01 | |  | |  | |  |
| Civil Status |  | |  | |  | |  | |  | | 4.07 | | 0.25 | |  |
| Partnered | 33 | | 16.67 | |  | | 31 | | 15.66 | |  | |  | |  |
| Unpartnered | 163 | | 82.32 | |  | | 165 | | 83.33 | |  | |  | |  |
| Other/category not listed | 2 | | 1.01 | |  | | 0 | | 0.00 | |  | |  | |  |
| Missing/declined to answer | 0 | | 0.00 | |  | | 2 | | 1.01 | |  | |  | |  |
| Living Arrangement | |  | |  | |  | |  | |  | | 2.31 | | 0.81 | |
| Alone | | 38 | | 19.19 | |  | | 40 | | 20.20 | |  | |  | |
| With family | | 105 | | 53.03 | |  | | 115 | | 58.08 | |  | |  | |
| With friend or roommate or pet | | 36 | | 18.18 | |  | | 28 | | 14.14 | |  | |  | |
| Supportive housing | | 12 | | 6.06 | |  | | 8 | | 4.04 | |  | |  | |
| Other/category not listed | | 6 | | 3.03 | |  | | 6 | | 3.03 | |  | |  | |
| Missing/declined to answer | | 1 | | 0.51 | |  | | 1 | | 0.51 | |  | |  | |
| Government Assistance^a^ | |  | |  | |  | |  | |  | | 10.41 | | 0.32 | |
| Unemployment | | 11 | | 5.56 | |  | | 10 | | 5.05 | |  | |  | |
| Medicare | | 32 | | 16.16 | |  | | 29 | | 14.65 | |  | |  | |
| Medicaid | | 60 | | 30.30 | |  | | 69 | | 34.85 | |  | |  | |
| Social Security | | 25 | | 12.63 | |  | | 30 | | 15.15 | |  | |  | |
| Food Stamps | | 54 | | 27.27 | |  | | 59 | | 29.80 | |  | |  | |
| SSI/SSDI | | 44 | | 22.22 | |  | | 41 | | 20.71 | |  | |  | |
| SNAP | | 17 | | 8.59 | |  | | 6 | | 3.03 | |  | |  | |
| None | | 2 | | 1.01 | |  | | 4 | | 2.02 | |  | |  | |
| Other/category not listed | | 38 | | 19.19 | |  | | 28 | | 14.14 | |  | |  | |
| Missing/declined to answer | | 38 | | 19.19 | |  | | 49 | | 24.75 | |  | |  | |
| Annual Personal Income | |  | |  | |  | |  | |  | | 5.75 | | 0.57 | |
| <$10,000 | | 60 | | 30.30 | |  | | 57 | | 28.79 | |  | |  | |
| $10,000-$20,000 | | 47 | | 23.74 | |  | | 57 | | 28.79 | |  | |  | |
| $20,000-$30,000 | | 18 | | 9.09 | |  | | 20 | | 10.10 | |  | |  | |
| $30,00-$40,000 | | 15 | | 7.58 | |  | | 8 | | 4.04 | |  | |  | |
| $40,000-$50,000 | | 4 | | 2.02 | |  | | 6 | | 3.03 | |  | |  | |
| >= $50,000 | | 16 | | 8.08 | |  | | 9 | | 4.55 | |  | |  | |
| I don’t know my income | | 37 | | 18.69 | |  | | 40 | | 20.02 | |  | |  | |
| Missing/declined to answer | | 1 | | 0.51 | |  | | 1 | | 0.51 | |  | |  | |
| Annual Household income | |  | |  | |  | |  | |  | | 3.15 | | 0.87 | |
| <$10,000 | | 39 | | 19.70 | |  | | 33 | | 16.67 | |  | |  | |
| $10,000-$20,000 | | 40 | | 20.20 | |  | | 47 | | 23.74 | |  | |  | |
| $20,000-$30,000 | | 17 | | 8.59 | |  | | 21 | | 10.61 | |  | |  | |
| $30,00-$40,000 | | 14 | | 7.07 | |  | | 10 | | 5.05 | |  | |  | |
| $40,000-$50,000 | | 5 | | 2.53 | |  | | 8 | | 4.04 | |  | |  | |
| >= $50,000 | | 28 | | 14.14 | |  | | 24 | | 12.12 | |  | |  | |
| I don’t know my income | | 52 | | 26.26 | |  | | 52 | | 26.26 | |  | |  | |
| Missing/declined to answer | | 3 | | 1.52 | |  | | 3 | | 1.52 | |  | |  | |
| Self-reported diagnosis^b^ |  | |  | |  | |  | |  | | 7.20 | | 0.84 | |  |
| Neurodevelopmental disorders | 19 | | 9.60 | |  | | 17 | | 8.59 | |  | |  | |  |
| Psychosis | 67 | | 33.84 | |  | | 63 | | 31.82 | |  | |  | |  |
| Bipolar Disorder | 42 | | 21.21 | |  | | 51 | | 25.76 | |  | |  | |  |
| Major Depressive Disorder | 88 | | 44.44 | |  | | 97 | | 48.99 | |  | |  | |  |
| Anxiety disorders | 109 | | 55.05 | |  | | 111 | | 56.06 | |  | |  | |  |
| Obsessive-compulsive and related disorders | 8 | | 4.04 | |  | | 8 | | 4.04 | |  | |  | |  |
| Trauma and stressor-related disorders | 51 | | 25.76 | |  | | 59 | | 29.8 | |  | |  | |  |
| Dissociative disorders | 2 | | 1.01 | |  | | 4 | | 2.02 | |  | |  | |  |
| Personality disorders | 8 | | 4.04 | |  | | 6 | | 3.03 | |  | |  | |  |
| Feeding and eating disorders | 0 | | 0.00 | |  | | 4 | | 2.02 | |  | |  | |  |
| Substance-related and addictive disorders | 3 | | 1.52 | |  | | 3 | | 1.52 | |  | |  | |  |
| Other/category not listed | 4 | | 2.02 | |  | | 4 | | 2.02 | |  | |  | |  |
| Missing/declined to answer | 14 | | 7.07 | |  | | 10 | | 5.05 | |  | |  | |  |
|  |  | |  | |  | |  | |  | |  | |  | |  |
|  | ***Mean*** | | ***SD*** | |  | | ***Mean*** | | ***SD*** | | **T** | | ***p*-value** | |  |
| Age | 43.73 | | 15.94 | |  | | 41.08 | | 15.43 | | 1.67 | | 0.10 | |  |
| Education (years) | 14.39 | | 3.54 | |  | | 13.76 | | 3.17 | | 1.84 | | 0.07 | |  |

*Note.* Chi-squared was used for categorical variables, and *t* tests were used for continuous variables. ^a^ Some patients endorsed more than one government assistance category ^b^Comorbidity was common.

**Supplement Table 6. Aim 3: Mediation Models of Acceptability, Feasibility, and Appropriateness on Relations between TSC condition (Standard vs. Adapted) and Outcome at POST and 6FU**

|  | coefficient | SE | z | *p* | 95% Confidence Interval of effect | %MP |
| --- | --- | --- | --- | --- | --- | --- |
| **Aim 3 Model 1: TSC 🡪 Acceptability 🡪 PROMIS-SD POST** | | | | | |  |
| Path a | -0.02 | 0.25 | -0.07 | 0.95 | -0.50, 0.46 | - |
| Path b | -9.96 | 5.46 | -1.83 | 0.07 | -20.65, 0.74 | - |
| Total effect | -13.87 | 6.01 | -2.31 | 0.02 | -25.65, -2.09 | - |
| Indirect effect | 0.17 | 2.44 | 0.07 | 0.95 | -4.62, 4.95 | 1.23% |
| **Aim 3 Model 2: TSC 🡪 Acceptability 🡪 PROMIS-SD 6FU** | | | | | |  |
| Path a | -0.02 | 0.25 | -0.07 | 0.95 | -0.50, 0.46 | - |
| Path b | -5.11 | 2.10 | -2.43 | 0.02 | -9.24, -0.99 | - |
| Total effect | -2.88 | 2.45 | -1.17 | 0.24 | -7.69, 1.93 | - |
| Indirect effect | 0.09 | 1.26 | 0.07 | 0.95 | -2.39, 2.56 | 3.13% |
| **Aim 3 Model 3: TSC 🡪 Acceptability 🡪 PROMIS-SRI POST** | | | | | |  |
| Path a | -0.02 | 0.22 | -0.10 | 0.92 | -0.45, 0.41 | - |
| Path b | -12.34 | 5.48 | -2.25 | 0.02 | -23.08, -1.59 | - |
| Total effect | -11.96 | 5.97 | -2.00 | 0.05 | -23.67, -0.25 |  |
| Indirect effect | 0.26 | 2.70 | 0.10 | 0.92 | -5.04, 5.56 | 2.17% |
| **Aim 3 Model 4: TSC 🡪 Acceptability 🡪 PROMIS-SRI 6FU** | | | | | |  |
| Path a | -0.02 | 0.22 | -0.10 | 0.92 | -0.45, 0.41 | - |
| Path b | -4.89 | 2.98 | -1.64 | 0.10 | -10.74, 0.95 | - |
| Total effect | -7.09 | 3.29 | -2.16 | 0.03 | -13.52, -0.65 | - |
| Indirect effect | 0.10 | 1.09 | 0.09 | 0.92 | -2.04, 2.24 | 1.41% |
| **Aim 3 Model 5: TSC 🡪 Acceptability 🡪 DSM-5 Cross-Cutting POST** | | | | | |  |
| Path a | -0.01 | 0.23 | -0.04 | 0.97 | -0.46, 0.44 | - |
| Path b | -4.56 | 2.50 | -1.82 | 0.07 | -9.47, 0.34 | - |
| Total effect | -6.42 | 3.87 | -1.66 | 0.10 | -14.02, 1.16 | - |
| Indirect effect | 0.05 | 1.04 | 0.04 | 0.97 | -1.99, 2.08 | 0.78% |
| **Aim 3 Model 6: TSC 🡪 Acceptability 🡪 DSM-5 Cross-Cutting 6FU** | | | | | |  |
| Path a | -0.01 | 0.23 | -0.04 | 0.97 | -0.46, 0.44 | - |
| Path b | -0.39 | 1.63 | -0.24 | 0.81 | -3.58, 2.80 | - |
| Total effect | 0.56 | 1.86 | 0.30 | 0.76 | -3.09, 4.21 | - |
| Indirect effect | 0.004 | 0.09 | 0.04 | 0.97 | -0.18, 0.19 | 0.71% |
| **Aim 3 Model 7: TSC 🡪 Acceptability 🡪 SDS POST** | | | | | | |
| Path a | -0.02 | 0.21 | -0.08 | 0.94 | -0.43, 0.40 | - |
| Path b | -4.44 | 3.08 | -1.44 | 0.15 | -10.48, 1.59 | - |
| Total effect | -3.41 | 2.36 | -1.45 | 0.15 | -8.02, 1.21 | - |
| Indirect effect | 0.07 | 0.94 | 0.08 | 0.94 | -1.78, 1.92 | 2.05% |
| **Aim 3 Model 8: TSC 🡪 Acceptability 🡪 SDS 6FU** | | | | | | |
| Path a | -0.02 | 0.21 | -0.08 | 0.94 | -0.43, 0.40 | - |
| Path b | -2.86 | 1.72 | -1.67 | 0.10 | -6.23, 0.51 | - |
| Total effect | -3.03 | 2.47 | -1.23 | 0.22 | -7.87, 1.80 | - |
| Indirect effect | 0.05 | 0.61 | 0.08 | 0.94 | -1.16, 1.25 | 1.65% |
| **Aim 3 Model 9: TSC 🡪 Appropriateness 🡪 PROMIS-SD POST** | | | | | | |
| Path a | 0.02 | 0.16 | 0.15 | 0.88 | -0.29, 0.34 | - |
| Path b | -6.86 | 4.67 | -1.47 | 0.14 | -16.02, 2.30 | - |
| Total effect | -10.19 | 4.90 | -2.08 | 0.04 | -19.80, -0.59 | - |
| Indirect effect | -0.16 | 1.10 | -0.15 | 0.88 | -2.31, 1.98 | 1.57% |
| **Aim 3 Model 10: TSC 🡪 Appropriateness 🡪 PROMIS-SD 6FU** | | | | | | |
| Path a | 0.02 | 0.16 | 0.15 | 0.88 | -0.29, 0.34 | - |
| Path b | -5.27 | 2.18 | -2.42 | 0.02 | -9.54, -1.01 | - |
| Total effect | -1.27 | 2.61 | -0.49 | 0.63 | -6.38, 3.84 | - |
| Indirect effect | -0.13 | 0.85 | -0.15 | 0.88 | -1.80, 1.54 | 10.24% |
| **Aim 3 Model 11: TSC 🡪 Appropriateness 🡪 PROMIS-SRI POST** | | | | | | |
| Path a | 0.04 | 0.14 | 0.28 | 0.78 | -0.24, 0.32 | - |
| Path b | -9.85 | 4.44 | -2.22 | 0.03 | -18.55, -1.15 | - |
| Total effect | -6.75 | 3.84 | -1.76 | 0.08 | -14.28, 0.78 | - |
| Indirect effect | -0.39 | 1.38 | -0.28 | 0.78 | -3.10, 2.31 | 5.78% |
| **Aim 3 Model 12: TSC 🡪 Appropriateness 🡪 PROMIS-SRI 6FU** | | | | | | |
| Path a | 0.04 | 0.14 | 0.28 | 0.78 | -0.24, 0.32 | - |
| Path b | -7.53 | 2.83 | -2.66 | 0.01 | -13.07, -1.98 | - |
| Total effect | -4.16 | 3.02 | -1.38 | 0.17 | -10.08, 1.77 | - |
| Indirect effect | -0.30 | 1.05 | -0.29 | 0.78 | -2.35, 1.75 | 7.21% |
| **Aim 3 Model 13: TSC 🡪 Appropriateness 🡪 DSM-5 POST** | | | | | | |
| Path a | 0.04 | 0.15 | 0.26 | 0.79 | -0.26, 0.34 | - |
| Path b | 0.46 | 2.71 | 0.17 | 0.86 | -4.86, 5.79 | - |
| Total effect | -3.64 | 3.13 | -1.16 | 0.25 | -9.78, 2.50 | - |
| Indirect effect | 0.02 | 0.15 | 0.13 | 0.90 | -0.27, 0.30 | 0.55% |
| **Aim 3 Model 14: TSC 🡪 Appropriateness 🡪 DSM-5 6FU** | | | | | | |
| Path a | 0.04 | 0.15 | 0.26 | 0.79 | -0.26, 0.34 | - |
| Path b | 0.18 | 2.18 | 0.08 | 0.94 | -4.10, 4.46 | - |
| Total effect | 1.10 | 1.88 | 0.58 | 0.56 | -2.59, 4.79 | - |
| Indirect effect | 0.01 | 0.10 | 0.07 | 0.94 | -0.19, 0.20 | 0.91% |
| **Aim 3 Model 15: TSC 🡪 Appropriateness 🡪 SDS POST** | | | | | | |
| Path a | 0.05 | 0.14 | 0.39 | 0.70 | -0.22, 0.32 | - |
| Path b | 0.60 | 2.72 | 0.22 | 0.83 | -4.74, 5.94 | - |
| Total effect | -2.92 | 2.00 | -1.46 | 0.14 | -6.84, 0.99 | - |
| Indirect effect | 0.03 | 0.17 | 0.19 | 0.85 | -0.30, 0.37 | 1.03% |
| **Aim 3 Model 16: TSC 🡪 Appropriateness 🡪 SDS 6FU** | | | | | | |
| Path a | 0.05 | 0.14 | 0.39 | 0.70 | -0.22, 0.32 | - |
| Path b | -4.34 | 2.47 | -1.76 | 0.08 | -9.19, 0.50 | - |
| Total effect | -1.51 | 1.94 | -0.78 | 0.44 | -5.31, 2.29 | - |
| Indirect effect | -0.23 | 0.60 | -0.38 | 0.70 | -1.41, 0.95 | 15.23% |
| **Aim 3 Model 17: TSC 🡪 Feasibility 🡪 PROMIS-SD POST** | | | | | | |
| Path a | 0.06 | 0.24 | 0.27 | 0.79 | -0.41, 0.54 | - |
| Path b | -7.69 | 4.78 | -1.61 | 0.11 | -17.06, 1.68 | - |
| Total effect | -8.93 | 5.12 | -1.74 | 0.08 | -18.96, -1.11 | - |
| Indirect effect | -0.50 | 1.89 | -0.26 | 0.79 | -4.21, 3.21 | 5.60% |
| **Aim 3 Model 18: TSC 🡪 Feasibility 🡪 PROMIS-SD 6FU** | | | | | | |
| Path a | 0.06 | 0.24 | 0.27 | 0.79 | -0.41, 0.54 | - |
| Path b | -3.98 | 1.80 | -2.21 | 0.03 | -7.51, -0.45 | - |
| Total effect | -2.33 | 2.44 | -0.96 | 0.34 | -7.11, 2.45 | - |
| Indirect effect | -0.26 | 0.98 | -0.26 | 0.79 | -2.18, 1.66 | 11.16% |
| **Aim 3 Model 19: TSC 🡪 Feasibility 🡪 PROMIS-SRI POST** | | | | | | |
| Path a | 0.04 | 0.20 | 0.20 | 0.84 | -0.36, 0.44 | - |
| Path b | -10.50 | 4.92 | -2.13 | 0.03 | -20.14, -0.84 | - |
| Total effect | -6.10 | 4.28 | -1.42 | 0.15 | -14.49, 2.29 | - |
| Indirect effect | -0.43 | 2.16 | -0.20 | 0.84 | -4.66, 3.80 | 7.05% |
| **Aim 3 Model 20: TSC 🡪 Feasibility 🡪 PROMIS-SRI 6FU** | | | | | | |
| Path a | 0.04 | 0.20 | 0.20 | 0.84 | -0.36, 0.44 | - |
| Path b | -4.39 | 2.36 | -1.86 | 0.06 | -9.02, 0.24 | - |
| Total effect | -5.31 | 2.79 | -1.90 | 0.06 | -10.77, 0.16 | - |
| Indirect effect | -0.18 | 0.90 | -0.20 | 0.84 | -1.94, 1.58 | 3.39% |
| **Aim 3 Model 21: TSC 🡪 Feasibility 🡪 DSM-5 Cross-Cutting POST** | | | | | | |
| Path a | 0.06 | 0.22 | 0.26 | 0.80 | -0.37, 0.48 | - |
| Path b | -3.46 | 2.04 | -1.69 | 0.09 | -7.46, 0.54 | - |
| Total effect | -3.91 | 2.81 | -1.39 | 0.16 | -9.42, 1.59 | - |
| Indirect effect | -0.19 | 0.75 | -0.25 | 0.80 | -1.67, 1.28 | 4.86% |
| **Aim 3 Model 22: TSC 🡪 Feasibility 🡪 DSM-5 Cross-Cutting 6FU** | | | | | | |
| Path a | 0.06 | 0.22 | 0.26 | 0.80 | -0.37, 0.48 | - |
| Path b | -0.79 | 1.48 | -0.53 | 0.60 | -3.69, 2.11 | - |
| Total effect | 0.92 | 1.87 | 0.49 | 0.62 | -2.75, 4.60 | - |
| Indirect effect | -0.04 | 0.15 | -0.29 | 0.77 | -0.33, 0.25 | 4.35% |
| **Aim 3 Model 23: TSC 🡪 Feasibility 🡪 SDS POST** | | | | | | |
| Path a | 0.05 | 0.20 | 0.25 | 0.80 | -0.34, 0.43 | - |
| Path b | 0.25 | 2.26 | 0.11 | 0.91 | -4.18, 4.68 | - |
| Total effect | -2.92 | 1.97 | -1.48 | 0.14 | -6.79, 0.96 | - |
| Indirect effect | 0.01 | 0.12 | 0.10 | 0.92 | -0.23, 0.25 | 0.34% |
| **Aim 3 Model 24: TSC 🡪 Feasibility 🡪 SDS 6FU** | | | | | | |
| Path a | 0.05 | 0.20 | 0.25 | 0.80 | -0.33, 0.43 | - |
| Path b | -1.41 | 1.90 | -0.74 | 0.46 | -5.12, 2.31 | - |
| Total effect | -2.04 | 2.04 | -1.00 | 0.32 | -6.05, 1.97 | - |
| Indirect effect | -0.07 | 0.31 | -0.22 | 0.82 | -0.68, 0.54 | 3.43% |
| *Note.* "-" indicates that value is not relevant to model. SE = robust standard errors. %MP = mediated proportion (i.e., the proportion of the total effect that is explained by the indirect effect expressed as a percentage). TSC = TSC treatment condition (Standard versus Adapted). PROMIS-SD = PROMIS Sleep Disturbance. PROMIS-SRI = PROMIS Sleep-Related Impairment. DSM-5 = DSM-5 Cross-Cutting. SDS = Sheehan Disability Scale. POST = post-treatment assessment. 6FU = six-month follow-up assessment. Path a = path from the independent variable to mediator (i.e., TSC condition 🡪 acceptability, appropriateness, or feasibility). Path b = path from the mediator to the outcome (acceptability, appropriateness, or feasibility 🡪 PROMIS-SD, PROMIS-SRI, DSM-5 Cross-Cutting, or SDS). All models adjust for pre-treatment levels of the relevant mediator (i.e., acceptability, appropriateness, or feasibility) and relevant outcome (i.e., PROMIS-SD, PROMIS-SRI, DSM-5 Cross-Cutting, or SDS). | | | | | | |

**Supplement Table 7. Means, Standard Deviations, and Effect Sizes for Patient Exploratory Outcomes**

|  | Pre-Treatment | | | | Post-Treatment | | | | 6FU | | | | |  |  |
| --- | --- | --- | --- | --- | --- | --- | --- | --- | --- | --- | --- | --- | --- | --- | --- |
|  | **Standard** | | **Adapted** | | **Standard** | | **Adapted** | | **Standard** | | | **Adapted** | | ***d pre-post*** | ***d pre-6FU*** |
|  | **Mean** | **SD** | **Mean** | **SD** | **Mean** | **SD** | **Mean** | **SD** | | **Mean** | **SD** | **Mean** | **SD** |  |  |
| **Outcome** |  |  |  |  |  |  |  |  | |  |  |  |  |  |  |
| Suicidal Ideation Severity | 0.58 | 0.96 | 0.53 | 0.99 | 0.88 | 1.36 | 0.42 | 0.85 | | 0.67 | 1.14 | 0.52 | 1.11 | -0.42 | -0.10 |
| Mean Cigarettes Per Day | 8.83 | 7.38 | 11.18 | 8.3 | 6.40 | 10.57 | 10.93 | 8.21 | | 9.23 | 8.95 | 6.78 | 6.6 | 0.30 | -0.58 |
| Mean Caffeinated Drinks Per Day | 1.87 | 1.33 | 1.65 | 1.57 | 2.13 | 2.27 | 1.48 | 1.47 | | 2.31 | 2.73 | 1.61 | 1.54 | -0.30 | -0.36 |
| Mean Days of Alcohol Use in Past 30 Days | 2.18 | 5.31 | 2.25 | 5.26 | 1.88 | 6.49 | 1.76 | 4.75 | | 2.58 | 7.53 | 2.19 | 5.33 | -0.04 | -0.09 |
| Credibility | - | - | - | - | 7.81 | 1.25 | 7.79 | 1.61 | | - | - | - | - | - | - |
| Perceived Improvement | - | - | - | - | 68.33 | 26.15 | 62.63 | 29.32 | | - | - | - | - | - | - |
| *Note.* 6FU = six-month follow-up assessment. "-" means that the variable is not relevant at a given timepoint. ‘Standard’ and ‘Adapted’ indicate TSC condition. Effect sizes are represented with ‘*d*’ and were calculated following Feingold (2009, equation 5), using unadjusted change scores and raw standard deviations at pre-treatment from each treatment condition. Mean cigarettes per day was only reported by patients who endorsed smoking on ‘some days’ or ‘every day.’ | | | | | | | | | | | | | | | |

**Supplement Table 8. Exploratory Aim 1: Multilevel Modeling Results for TSC Condition (Adapted vs. Standard) on Suicidality and Cigarette, Caffeine, and Alcohol Use**

|  | **Post-Treatment** | | | **6FU** | | |
| --- | --- | --- | --- | --- | --- | --- |
|  | ***b*** | **SE** | ***p-*value** | ***b*** | **SE** | ***p-*value** |
| **Outcome** |  |  |  |  |  |  |
| SI Severity | -0.45 | 0.32 | 0.17 | -0.34 | 0.26 | 0.19 |
| Mean Cigarettes Per Day | 2.02 | 2.00 | 0.31 | -7.57 | 2.68 | **0.005** |
| Mean Caffeinated Drinks Per Day | 0.22 | 0.41 | 0.59 | -0.23 | 0.38 | 0.54 |
| Mean Days of Alcohol Use in Past 30 Days | -0.10 | 0.76 | 0.89 | 0.15 | 1.00 | 0.88 |
| *Note.* 6FU = six-month follow-up assessment. Bold indicates significant *p*-values. *b* = time-by-treatment interaction. SE = robust standard errors. Mean cigarettes per day was only reported by patients who endorsed smoking on ‘some days’ or ‘every day.’ | | | | | | |

**Supplement Table 9. Exploratory Aim 2: Multilevel Modeling Moderation Results for UC-DT versus TSC on Patient Outcomes from Pre- to Post-treatment**

|  | ***b*** | **SE** | ***p-*value** |
| --- | --- | --- | --- |
| **Moderator: Sex** |  |  |  |
| PROMIS-SD | -1.14 | 3.87 | 0.77 |
| PROMIS-SRI | -0.90 | 4.09 | 0.83 |
| SHC | 0.84 | 0.70 | 0.23 |
| DSM-5 | -2.29 | 2.89 | 0.43 |
| SDS | -0.61 | 2.69 | 0.82 |
| **Moderator: Age** |  |  |  |
| PROMIS-SD | 4.95 | 4.57 | 0.28 |
| PROMIS-SRI | -2.27 | 4.87 | 0.64 |
| SHC | -0.29 | 0.75 | 0.69 |
| DSM-5 | -3.49 | 3.44 | 0.31 |
| SDS | -0.69 | 3.08 | 0.82 |
| **Moderator: Baseline PROMIS-SD** | | |  |
| PROMIS-SD | - | - | - |
| PROMIS-SRI | -0.06 | 0.37 | 0.86 |
| SHC | 0.01 | 0.04 | 0.89 |
| DSM-5 | 0.07 | 0.20 | 0.73 |
| SDS | 0.10 | 0.17 | 0.54 |
| **Moderator: Baseline PROMIS-SRI** | | |  |
| PROMIS-SD | -0.01 | 0.26 | 0.96 |
| PROMIS-SRI | - | - | - |
| SHC | -0.06 | 0.05 | 0.24 |
| DSM-5 | -0.08 | 0.24 | 0.74 |
| SDS | -0.11 | 0.16 | 0.50 |
| **Moderator: Baseline DSM-5 Cross-Cutting** | | |  |
| PROMIS-SD | -0.10 | 0.25 | 0.69 |
| PROMIS-SRI | -0.29 | 0.25 | 0.26 |
| SHC | -0.02 | 0.04 | 0.66 |
| DSM-5 | - | - | - |
| SDS | -0.24 | 0.15 | 0.11 |
| Note. "-" means that a moderator/outcome pairing was not tested (i.e., when the moderator was already included as the outcome). *b* = time-by-treatment-by-moderator 3-way interaction. SE = robust standard errors. PROMIS-SD = PROMIS Sleep Disruption. PROMIS-SD = PROMIS Sleep Disturbance. PROMIS-SRI = PROMIS Sleep-Related Impairment. SHC = Sleep Health Composite (note, scored such that higher scores indicate better sleep health). DSM-5 = DSM-5 Cross-Cutting. SDS = Sheehan Disability Scale. | | | |

1. The site participating in Santa Clara is technically a federally qualified health center (FQHC), not a CMHC. Although there are some differences (e.g., funding structure, types of staff), these two types of sites are similar. Thus, for the purposes of this study, they are grouped together under “CMHC.” [↑](#footnote-ref-1)
